# Enzyme-loaded Fe^3+^-doped ZIF-90 particles as catalytic bioreactor hybrids for operating catalytic cascades†

**DOI:** 10.1039/d5sc01972a

**Published:** 2025-05-01

**Authors:** Jin Wang, Yunlong Qin, Raanan Carmieli, Vitaly Gutkin, Eli Pikarsky, Zhen Zhang, Xinghua Chen, Itamar Willner

**Affiliations:** a Institute of Chemistry, The Hebrew University of Jerusalem Jerusalem 91904 Israel itamar.willner@mail.huji.ac.il xinghua.chen@mail.huji.ac.il; b School of the Environment and Safety Engineering, Jiangsu University Zhenjiang 212013 China zhangzhen@ujs.edu.cn; c Department of Chemical Research Support, Weizmann Institute of Science Rehovot 76100 Israel; d The Center for Nanoscience and Nanotechnology, The Hebrew University of Jerusalem Jerusalem 91904 Israel; e Faculty of Medicine, The Hebrew University of Jerusalem Jerusalem 91120 Israel

## Abstract

Fe^3+^-doped ZIF-90 (Fe^3+^-ZIF-90), a metal–organic framework (MOF), was synthesized and characterized. The MOF particles reveal peroxidase-like activity reflected by catalyzing the H_2_O_2_ oxidation of 3,3′,5,5′-tetramethylbenzidine, TMB, to TMB˙^+^. Integration of the two enzymes, β-galactosidase, β-Gal, and glucose oxidase, GOx, in the Fe^3+^-ZIF-90 provides an organized framework allowing the operation of a three-catalyst cascade, where the β-Gal-catalyzed oxidation of lactose yields glucose and galactose, and the resulting glucose is aerobically oxidized by GOx to gluconic acid and H_2_O_2_, followed by the Fe^3+^-ZIF-90-catalyzed H_2_O_2_ oxidation of TMB to TMB˙^+^. The coupled bienzyme/nanozyme cascade in the MOFs is *ca.* 5-fold enhanced, as compared to a homogeneous mixture of the catalytic constituents. The enhanced catalytic activity of the enzyme cascades in the MOFs is attributed to the confined reaction framework, allowing product channeling across the multienzyme constituents and overcoming diffusion barriers. Moreover, the enzymes, acetylcholine esterase, AChE, and choline oxidase, ChOx, are encapsulated in the confined porous Fe^3+^-ZIF-90 particles. The catalytic cascade where the neurotransmitter acetylcholine is hydrolyzed by AChE followed by the stepwise ChOx-catalyzed oxidation of choline to betaine and H_2_O_2_, and the Fe^3+^-ZIF-90-catalyzed oxidation of TMB to colored TMB˙^+^ by H_2_O_2_ is demonstrated. The three-catalyst cascade is *ca.* 5-fold enhanced as compared to the mixture of separated catalysts. The integrated three-catalyst AChE/ChOx/Fe^3+^-ZIF-90 particles are applied as colorimetric sensors detecting the neurotransmitter acetylcholine and probing AChE inhibitors. The novelty of the systems is reflected by the assembly of multienzyme catalytic Fe^3+^-ZIF-90 hybrids in confined environments as bioreactor frameworks driving effective biocatalytic cascades.

## Abstract 2

Bienzyme biocatalytic cascades are coupled with Fe^3+^-doped ZIF-90 (Fe^3+^-ZIF-90) nanozymes to enable three-catalyst cascaded chemical transformations in confined media. The enzyme/nanozyme composites are applied as sensing platforms.

## Graphical abstract

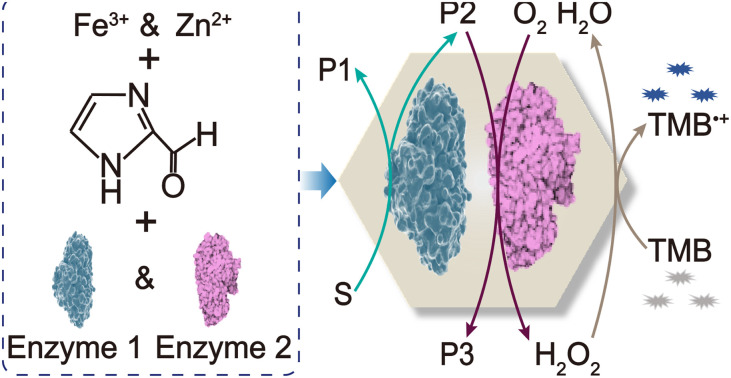

## Introduction

Metal–organic frameworks (MOFs) find growing interest as functional materials for catalysis,^1,2^ separation,^3,4^ gas storage,^5,6^ water capture,^7–9^ clean energy,^10^ food safety,^11,12^ and environment remediation.^13,14^ Their high loading capacity, high surface area, and chemical functionalization enable their diverse applications for analytical and medical uses, such as sensors,^15^ imaging,^16–18^ and drug carriers for controlled release.^19–22^

The catalytic functions of MOF particles can be engineered by three different strategies, including: (i) the use of the metal-ions integrated into the MOF as catalytic sites. For example, Cr^3+^ or Fe^3+^-MIL-100 MOF particles catalyzed the acetalization of benzaldehyde^23^ and Co^2+^-ZIF-67 catalyzed the transformation of epoxide into cyclic carbonate in the presence of CO_2_.^24^ (ii) The anchoring of auxiliary metal-ions or complexes to the ligands composing the MOFs. For example, ligation of Cu^2+^ to the bipyridine ligand associated with UiO-type MOFs yielded a peroxidase-like catalyst oxidizing NADH to NAD^+^ or dopamine to aminochrome by H_2_O_2_,^25^ and conjugation of a Cu^2+^–Schiff-base complex to an UiO-66–NH_2_(Zr) framework catalyzed the Knoevenagel condensation and Michael addition processes.^26^ (iii) The integration of homogeneous catalysts, catalytic nanoparticles/clusters or biocatalysts into the MOFs. For example, a Pd(ii)–bipyridine complex encapsulated in UiO-67 MOF particles catalyzed the Heck coupling reaction between aryl halides and olefins or the Suzuki–Miyaura coupling of aryl chlorides and aryl boronic acids.^27^ Also, Pd nanoparticles integrated into MIL-101–NH_2_ MOFs catalyzed the hydrogenation of nitroaromatic substrates,^28^ and gold nanoclusters loaded in UiO-66 catalyzed the oxidative esterification of furfural.^29^

The encapsulation of biomolecules within the MOF matrices is particularly interesting since it provides a means to stabilize the recyclable biocatalysts and enables the triggered release of biomolecule loads using stimuli-responsive MOFs. Nevertheless, since many of the MOFs are synthesized at high temperatures and organic solvents, the encapsulation of biomolecules in the MOF matrices during their synthesis is hampered by their thermal deactivation during the encapsulation process. An interesting sub-family of MOF particles, particularly as frameworks for encapsulation of biomolecules, is the family of zeolitic imidazolate framework (ZIF) particles.^30,31^ The biocompatibility of ZIF particles and their preparation in aqueous solution at room temperature make these particles ideal matrices for encapsulating biomolecules. Indeed, basic Zn^2+^–imidazolate frameworks (ZIF-8) provided a versatile matrix for the encapsulation of biomolecules, such as enzymes,^32–35^ antibodies,^36^ protein drugs (*e.g.*, insulin and vaccines),^37,38^ and DNA.^39,40^ Indeed, multienzymes were integrated into ZIF-8 matrices, and the advantages of the confined media on the cascaded catalysis were demonstrated.^33,41^ Nevertheless, the ZIF-8 matrices are not free of limitations. Their sensitivity to pH, degradability under acidic conditions, the lack of catalytic activities of the Zn^2+^ composing the frameworks, and the lack of surface functionalities allowing the further modification of the frameworks are serious limitations. Different strategies to overcome these difficulties were reported. These included the synthesis of ZIF particles composed of catalytic ions, *e.g.*, Co^2+^-ZIF-67,^42^ the stabilization of the ZIF structures by polymer coatings such as polyaniline,^43^ and the construction of catalytic ZIF structures with functionalized imidazolate ligands, such as carboxaldehyde-modified imidazolate that binds diamine tethers and stabilizes Au nanoparticles for catalyzed hydrogeneration of nitro-aromatic compounds.^44^ Also, Zn^2+^-ZIF composites were doped with catalytic metal ions, such as Fe^3+^, Cu^2+^ or Mn^2+^ and the resulting hybrid ZIF structures revealed diverse catalytic activities, such as the aerobic oxidation of benzylic hydrocarbon^45^ or cycloaddition reactions.^46^ Moreover, by tethering auxiliary catalysts, such as hemin/G-quadruplex, to carboxaldehyde groups associated with ZIF-90 units, the ZIF hybrid could guide catalytic reactions, *e.g.*, the catalyzed H_2_O_2_ oxidation of *N*-hydroxy-l-arginine to citrulline.^47^ Furthermore, structural changes induced by the ZIF particles on the encapsulated biocatalysts were reported to change the catalytic features of the catalytic loads.^48–50^ Different applications of the ZIF particles for sensing,^51,52^ controlled drug release^53,54^ and cascaded catalysis^33,41^ were demonstrated.

Here we wish to report the synthesis of Fe^3+^-doped ZIF-90 (Fe^3+^-ZIF-90) nanozymes exhibiting peroxidase-like catalytic functions and the assembly of multienzyme-loaded Fe^3+^-ZIF-90 MOFs. The biocatalyst/MOF composites are used as functional hybrids operating diverse catalytic cascades. Specifically, we integrate into the ZIF particles different single type enzymes, *e.g.*, glucose oxidase (GOx) or choline oxidase (ChOx), and coupled multienzymes, consisting of β-galactosidase (β-Gal)/GOx or acetylcholine esterase (AChE)/ChOx, which operate biocatalytic cascades. We describe the conjugation of the biocatalytic cascades to the peroxidase-like catalytic functions of the synthetic Fe^3+^-doped catalytic sites of the Fe^3+^-doped MOF particles. Beyond the novel coupling of biocatalytic cascades to synthetic catalytic sites of the MOF structures to operate a multi-modal catalytic bioreactor, we introduce the systems for sensing applications. Specifically, we address the use of the AChE/ChOx/Fe^3+^-ZIF-90 hybrid as a functional composite probing the activity of AChE, an important catalyst for metabolic degradation of the acetylcholine neurotransmitter. Furthermore, we introduce the use of the AChE/ChOx/Fe^3+^-ZIF-90 hybrid system to probe the effect of a 1,5-bis(4-allyldimethylammoniumphenyl)pentane-3-one dibromide (BW284C51) inhibitor, a nerve gas simulator, on the activity of AChE, thereby demonstrating the potential use of the system for sensitive detection of hazardous nerve gas. In the different coupled enzyme/nanozyme systems, we emphasize the importance of the confined reaction media of the frameworks on the coupled catalytic cascades by comparing the catalytic performance of the integrated assemblies to the catalytic cascades driven by the diffusive and separated enzyme/nanozyme constituents in bulk solution media.

## Results and discussion

**Fig. 1:**
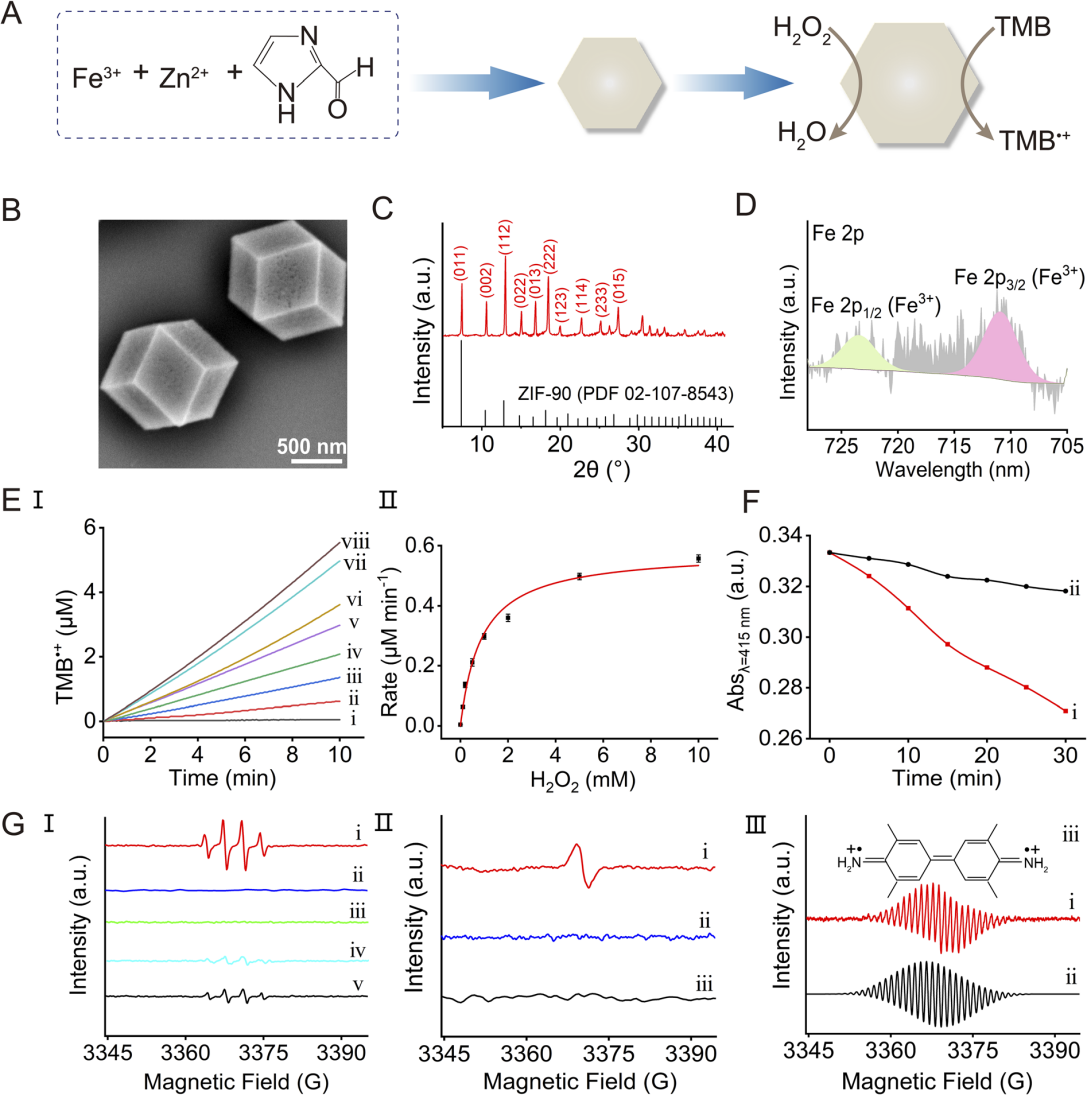
(A) Synthesis of the Fe^3+^-ZIF-90 framework and its schematic peroxidase-like function catalyzing the oxidation of TMB to TMB˙^+^ by H_2_O_2_. (B) SEM image of the Fe^3+^-ZIF-90 MOF particles. (C) XRD spectrum of the Fe^3+^-ZIF-90 particles: top—experimental results; bottom—spectral band of ZIF-90 from the PDF database. (D) Deconvoluted Fe 2p XPS spectra of the Fe^3+^-ZIF-90 MOF particles. (E) Panel I: time-dependent concentration changes of TMB˙^+^ using TMB, 2 mM, and the Fe^3+^-ZIF-90 catalyst, 100 μg mL^−1^, in the presence of variable concentrations of H_2_O_2_: (i) 0 mM; (ii) 0.1 mM; (iii) 0.2 mM; (iv) 0.5 mM; (v) 1 mM; (vi) 2 mM; (vii) 5 mM; (viii) 10 mM. Panel II: rates of TMB˙^+^ formation in the presence of Fe^3+^-ZIF-90, 100 μg mL^−1^, TMB, 2 mM, and variable concentrations of H_2_O_2_. (F) Time-dependent absorbance changes of DPBF, 60 μM, as a result of ROS agent formation: (i) in the presence of Fe^3+^-ZIF-90, 100 μg mL^−1^, and H_2_O_2_, 10 mM; (ii) in the presence of Fe^3+^-ZIF-90 particles, 100 μg mL^−1^, and in the absence of H_2_O_2_. (G) EPR spectra corresponding to: panel I—(i) ˙OH radicals generated by Fe^3+^-ZIF-90 and H_2_O_2_; (ii) and (iii) control systems consisting of only Fe^3+^-ZIF-90 and only H_2_O_2_; (iv) ˙OH generated by Fe^3+^-ZIF-90/H_2_O_2_ and quenched by dimethyl sulfoxide; (v) ˙OH generated by the Fe^3+^-ZIF-90/H_2_O_2_ system quenched by TMB. In all experiments ˙OH is trapped by BMPO. Panel II—(i) the spectrum of TMB˙^+^ formed in the presence of Fe^3+^-ZIF-90/H_2_O_2_ and TMB; (ii) and (iii) control systems demonstrating the lack of TMB˙^+^ in systems composed of only Fe^3+^-ZIF-90/TMB or H_2_O_2_/TMB. Panel III—high resolution spectra of TMB˙^+^: (i) generated by the Fe^3+^-ZIF-90/H_2_O_2_/TMB system; (ii) computationally simulated TMB˙^+^; (iii) the chemical structure of TMB˙^+^.

The Fe^3+^-ZIF-90 MOFs were prepared according to the reported method,^55^ with slight modification, by mixing Zn(NO_3_)_2_ and FeCl_3_ with 2-imidazolecarboxaldehyde and polyvinylpyrrolidone in a water/ethanol solution and stirring the mixture at room temperature, as shown in Fig. 1A. The particles, *ca.* 1 μm in diameter, exhibit a rhombic dodecahedral structure, as shown in Fig. 1B. The powder X-ray diffraction (PXRD) spectrum of the particles, as shown in Fig. 1C, overlaps the spectrum of ZIF-90 (PDF 02-107-8543), indicating that the crystalline structure of ZIF-90 is retained in the doped particles. The deconvoluted Fe 2p X-ray photoelectron spectroscopy (XPS) spectra in Fig. 1D indicate that the doped iron exists in the Fe^3+^-state (for the XPS spectra of the other elements included in the particles, see Fig. S1†). The inductively coupled plasma mass spectrometry (ICP-MS) measurement indicates a *ca.* 5% loading of the doped ions (170.7 μg mg^−1^ of Zn^2+^; 7.3 μg mg^−1^ of Fe^3+^). The N_2_ adsorption/desorption measurement (Fig. S2†) reveals a Brunauer–Emmett–Teller (BET) specific surface area of 317.65 m^2^ g^−1^.

The Fe^3+^-ZIF-90 particles demonstrate peroxidase-like activity reflected by the catalyzed H_2_O_2_ oxidation of 3,3′,5,5′-tetramethylbenzidine, TMB, generating the blue-colored oxidized TMB, TMB˙^+^, as shown in Fig. 1E. In addition, the Fe^3+^-ZIF-90 demonstrates other peroxidase-like activities, such as NADH peroxidase function, as shown in Fig. S3.† The reactive oxygen species (ROS) generated by the Fe^3+^-ZIF-90 particles in the presence of H_2_O_2_ and their participation in the oxidation of TMB were characterized spectroscopically, revealing the formation of hydroxyl radicals (˙OH), as shown in Fig. 1F, G and S4.†Fig. 1F and S4† depict the effective temporal depletion of the absorbance of 1,3-diphenylisobenzofuran (DPBF) by the ROS generated in the presence of both Fe^3+^-ZIF-90 and H_2_O_2_ as compared to the low absorbance changes in the presence of the Fe^3+^-ZIF-90 and in the absence of H_2_O_2_. Fig. 1G displays electron paramagnetic resonance (EPR) spectra using 5-*tert*-butoxycarbonyl-5-methyl-1-pyrroline-*N*-oxide (BMPO) as the trapping agent, confirming the catalyzed formation of ˙OH as ROS products and their participation in the oxidation of TMB. In panel I, the characteristic ˙OH spectrum, in the presence of both Fe^3+^-ZIF-90 particles and H_2_O_2_, curve (i), is observed while control experiments show that no radicals are formed in the presence of particles and in the absence of H_2_O_2_, curve (ii), and in the presence of H_2_O_2_ and in the absence of MOFs, curve (iii). Panel I further reveals the formation of ˙OH by the quenching effect of dimethyl sulfoxide, curve (iv), and their participation in the oxidation of TMB, curve (v). Furthermore, the reaction of ˙OH with TMB is confirmed by the formation of TMB˙^+^, panel II. The high resolution EPR spectrum of TMB˙^+^ (generated in the absence of BMPO) is depicted in panel III, curve (i), and the computationally simulated spectrum is displayed in curve (ii). The results are consistent with the reported spectrum of TMB˙^+^, and the computational spectrum allows the mapping of the electron distribution in the molecular framework,^56^ as presented in the inset structure (iii) (for the mechanistic path corresponding to the Fe^3+^-ZIF-90 catalyzed generation of ˙OH, see ESI P. S11†).

**Fig. 2:**
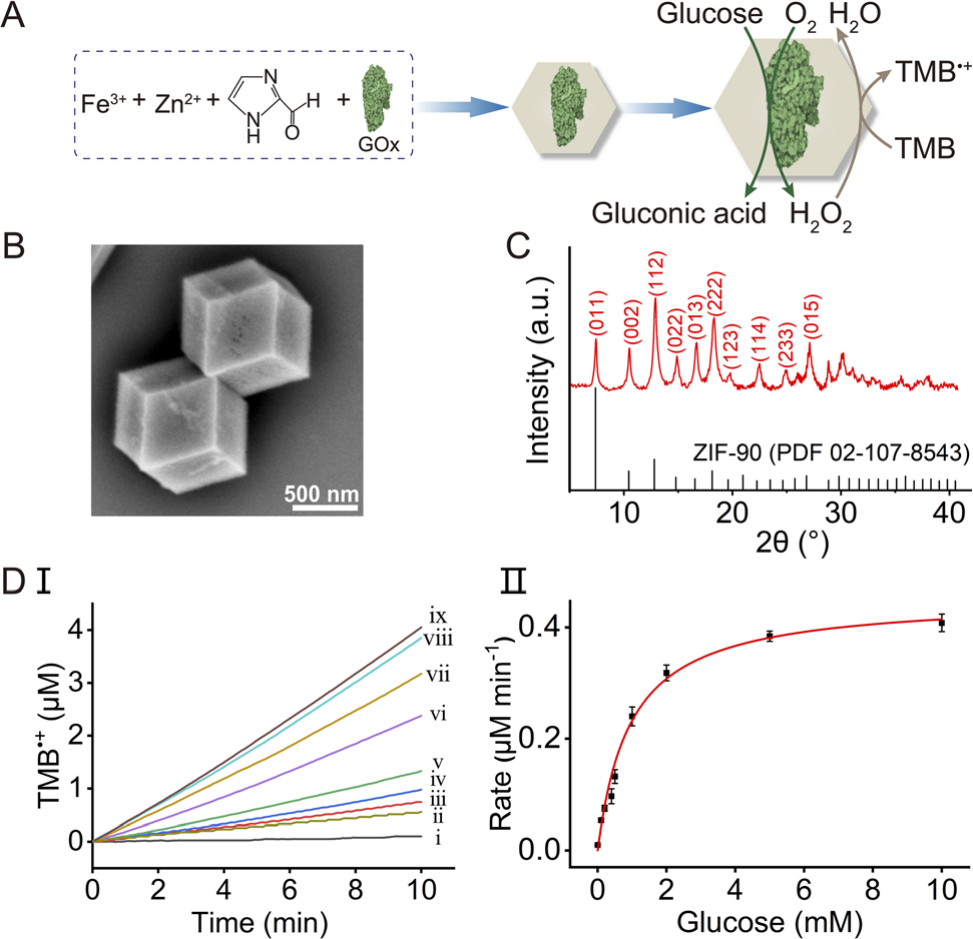
(A) Schematic synthesis of GOx-loaded Fe^3+^-ZIF-90 particles and the bioreactor activities towards the cascaded GOx-catalyzed aerobic oxidation of glucose, followed by the Fe^3+^-ZIF-90-catalyzed oxidation of TMB to TMB˙^+^ by the generated H_2_O_2_. (B) SEM image of GOx-loaded Fe^3+^-ZIF-90 particles. (C) XRD spectrum of the GOx-loaded Fe^3+^-ZIF-90 particles: top—experimental results; bottom—spectral band from the PDF database. (D) Panel I: time-dependent concentration changes of TMB˙^+^ upon operating the GOx-loaded Fe^3+^-ZIF-90 framework, 100 μg mL^−1^, and TMB, 2 mM, in the presence of variable concentrations of glucose: (i) 0 mM; (ii) 0.1 mM; (iii) 0.2 mM; (iv) 0.4 mM; (v) 0.5 mM; (vi) 1 mM; (vii) 2 mM; (viii) 5 mM; (ix) 10 mM. Panel II: rates of TMB oxidation to TMB˙^+^ by the GOx-loaded Fe^3+^-ZIF-90/TMB system as a function of glucose concentration.

The intrinsic peroxidase activities of the Fe^3+^-doped ZIF-90 frameworks were then implemented to operate the MOF-mediated biocatalytic cascade by integrating enzymes in the Fe^3+^-ZIF-90 frameworks. In the first step, GOx was integrated into the frameworks during the course of the particle synthesis, as shown in Fig. 2A. Two methods were employed to estimate the loading of the enzyme in the particles: (i) the residual content of GOx in the solution after encapsulation of the enzyme in the particles was evaluated using the GOx/horseradish peroxidase (HPR) standard probing cascade and an appropriate calibration curve (see Fig. S5† and accompanying discussion for details). The loading of GOx in the Fe^3+^-ZIF-90, using this method, was estimated to be 97.4 μg mg^−1^. (ii) GOx was modified with fluorescein isothiocyanate, FITC. As shown in Fig. S6,† panels I–III display the fluorescence confocal microscopy image of the FITC-modified GOx integrated into the particles. Using a calibration curve relating the fluorescence intensity of FITC–GOx at different concentrations of the labeled enzyme (Fig. S7A and B†) and probing the fluorescence intensity of residual content of the enzyme in solution after encapsulation in the framework (Fig. S7C†), the loading of the fluorescent GOx in the particles was evaluated to be 94.2 μg mg^−1^ particles, in good agreement with the loading determined by method (i). The GOx-loaded Fe^3+^-ZIF-90 particles were characterized by SEM (Fig. 2B) and PXRD (Fig. 2C) demonstrating that the morphology and crystallinity of the ZIF-90 were retained after integration of the enzyme into the framework.

The GOx-loaded Fe^3+^-ZIF-90 MOFs were then implemented as a catalytic reactor for the cascaded glucose-driven oxidation of TMB, as shown in Fig. 2A. In this system, the GOx-catalyzed aerobic oxidation of glucose yields gluconic acid and H_2_O_2_, and the Fe^3+^-ZIF-90 catalyzes the oxidation of TMB to TMB˙^+^ by the generated H_2_O_2_. As shown in Fig. 2D, panel I presents the time-dependent concentration changes of TMB˙^+^ generated by the integrated frameworks in the presence of variable concentrations of glucose. The rate curve corresponding to the GOx-integrated system is displayed in panel II, and the respective *V*_max_ = 0.45 μM min^−1^ was derived. In addition, Fig. S8† shows the time-dependent concentration changes of TMB˙^+^, in the presence of glucose (5 mM), using different amounts of GOx loads in the integrated frameworks. As the loading amount increases, the glucose-driven catalytic oxidation of TMB to TMB˙^+^ by the GOx-integrated system is enhanced.

**Fig. 3:**
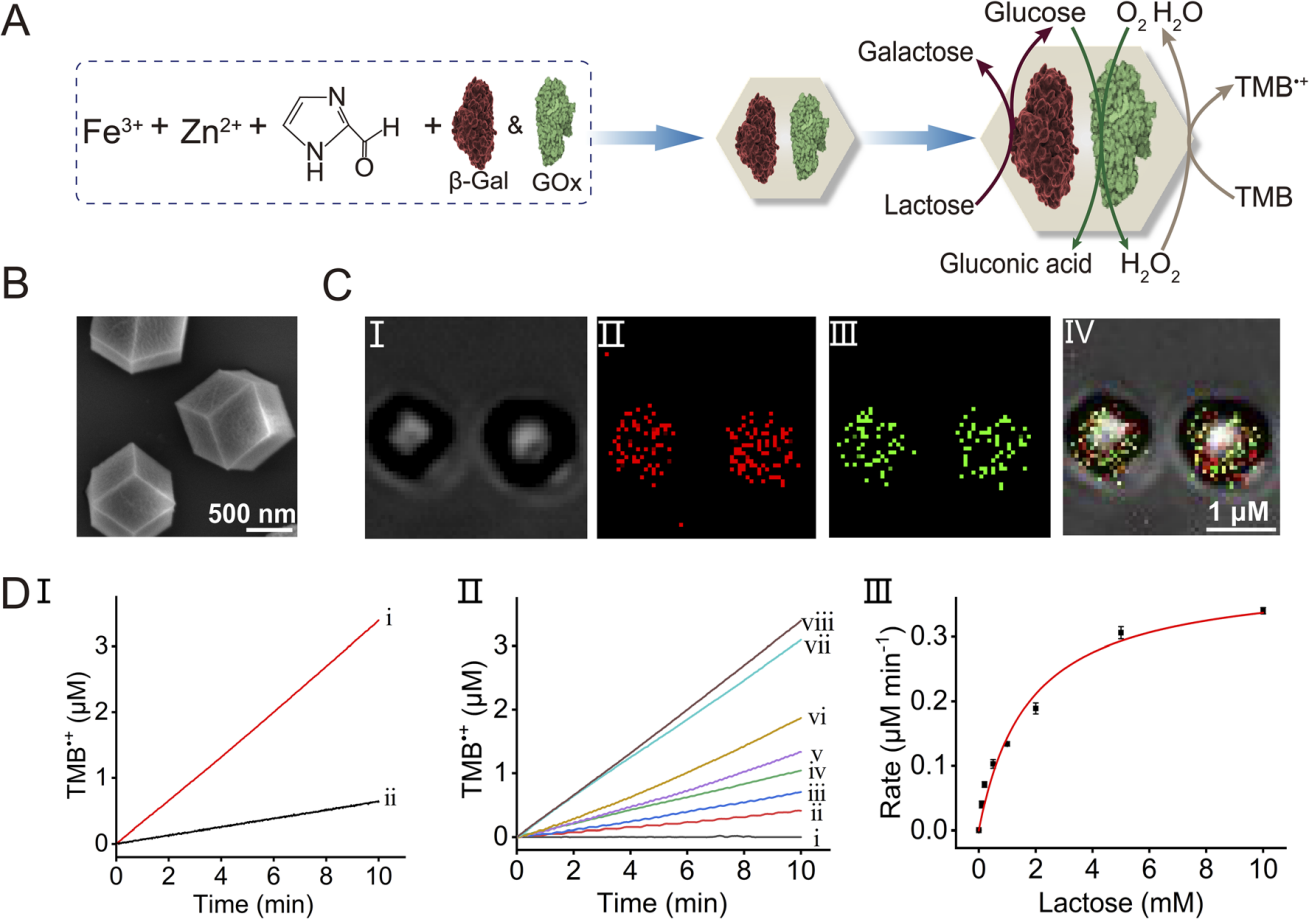
(A) Schematic assembly of the two enzymes (β-Gal and GOx) into the porous Fe^3+^-ZIF-90 MOF particles and their use as a three-catalyst cascade driving the β-Gal-catalyzed hydrolysis of lactose, the subsequent aerobic oxidation of glucose, and the cascaded Fe^3+^-ZIF-90-catalyzed oxidation of TMB by H_2_O_2_ generated by the biocatalytic cascade. (B) SEM image of the β-Gal/GOx-loaded Fe^3+^-ZIF-90 MOFs. (C) Bright field and fluorescence confocal microscopy images corresponding to the Atto 565-labeled β-Gal/FITC-labeled GOx loaded in the Fe^3+^-ZIF-90: panel I—bright field image; panel II—imaging the Atto 565-labeled β-Gal (*λ*_ex_ = 561 nm, *λ*_em_ = 585 nm); panel III—imaging FITC-labeled GOx (*λ*_ex_ = 488 nm, *λ*_em_ = 525 nm); panel IV—overlapped image. (D) Panel I—time-dependent concentration changes of TMB˙^+^ upon operating the lactose-driving three-catalyst cascade using: (i) the β-Gal/GOx-loaded Fe^3+^-ZIF-90 MOFs as an integrated assembly; (ii) the separated β-Gal, GOx and Fe^3+^-ZIF-90 constituents in the homogeneous aqueous phase at concentrations identical to those in (i). Panel II—time-dependent concentration changes of TMB˙^+^ upon operating the β-Gal/GOx/Fe^3+^-ZIF-90 cascade in the presence of variable concentrations of lactose: (i) 0 mM; (ii) 0.1 mM; (iii) 0.2 mM; (iv) 0.5 mM; (v) 1 mM; (vi) 2 mM; (vii) 5 mM; (viii) 10 mM. Panel III—rates of TMB˙^+^ formation catalyzed by the β-Gal/GOx/Fe^3+^-ZIF-90 system in the presence of variable concentrations of lactose.

The advantages of operating catalytic cascades in confined reaction media, as compared to diffusionally operating enzyme cascades, attracted substantial research interest as a means of mimicking native enzyme cascades in biologically confined environments,^57^*e.g.*, cell. The spatial proximity of the catalysts in the confined media overcomes diffusional barriers of the substrates/products tunneling between the catalysts participating in the catalytic cascade by providing directional and effective interconnective communication between the catalysts. Indeed, diverse biocatalytic cascades operating in confined media, such as tethered enzymes on programmed DNA nanostructures,^58–61^ encapsulation of enzymes in microdroplets^62,63^ or polymersomes,^64^ demonstrated the advantages of operating the biocatalytic cascades in the organized assemblies. The ability of integrating multienzyme agents in the porous metal–organic frameworks and particularly the feasibility to couple the biocatalytic cascade to the peroxidase-like catalytic functions of the Fe^3+^-ZIF-90 particles, provide a versatile means to engineer new biocatalyst/Fe^3+^-ZIF-90 composite reactors demonstrating the advantages of the confined porous particles as a hybrid reactor system driving the catalytic system. This is exemplified in Fig. 3A with the integration of two enzymes, β-Gal and GOx, in the Fe^3+^-ZIF-90 porous particles. This bienzyme/Fe^3+^-ZIF-90 composite allows, in principle, to drive the three-catalyst cascade in the confined frameworks, where β-Gal acts as a primary biocatalyst for the catalyzed hydrolysis of lactose to galactose and glucose. The glucose product is then tunneled to GOx as a second enzyme, catalyzing the aerobic oxidation of glucose to gluconic acid and H_2_O_2_. The resulting H_2_O_2_ is then transferred to the porous Fe^3+^-ZIF-90 nanozyme that catalyzes the oxidation of TMB to TMB˙^+^. Accordingly, the two enzymes, β-Gal and GOx, were integrated into the Fe^3+^-ZIF-90 frameworks within the process of the formation of the particles (see the ESI Experimental section† for details). Fig. 3B depicts the SEM image of the resulting bienzyme-loaded particles, demonstrating that the rhombic dodecahedral crystallinity of the ZIF-90 is retained. The successful entrapment of the enzymes in the Fe^3+^-ZIF-90 particles was confirmed by quantitative evaluation of the enzyme loading in the frameworks and by demonstrating the operation of the catalytic cascade by the particles (*vide infra*). Towards this goal, β-Gal was labeled with the Atto 565 fluorophore (*λ*_ex_ = 561 nm, *λ*_em_ = 585 nm) and GOx was labeled with FITC (*λ*_ex_ = 488 nm, *λ*_em_ = 525 nm). Fig. 3C depicts the bright field, fluorescence confocal microscopy and merged images of the particles, demonstrating that the two enzymes were, indeed, integrated into the Fe^3+^-ZIF-90 particles. By monitoring the fluorescence of the residual enzymes in the solution after preparing the particles (and knowing the initial concentrations of the enzymes) and employing appropriate calibration curves relating the fluorescence intensities of the fluorophore-labeled enzyme to their contents (Fig. S7, S9 and S10†), the loading of the enzymes in the particles was evaluated. The loading of β-Gal and GOx was determined to be 82.6 μg mg^−1^ and 96.1 μg mg^−1^, respectively. The β-Gal/GOx-loaded Fe^3+^-ZIF-90 particles were then applied to activate the three-catalyst cascade. Fig. 3D, panel I shows the time-dependent concentration changes of TMB˙^+^ originating upon operation of the three-catalyst cascaded frameworks, β-Gal/GOx/Fe^3+^-ZIF-90, in the presence of lactose, 10 mM (curve (i)). For comparison, the formation of TMB˙^+^ by the diffusional mixture consisting of separated β-Gal, GOx and Fe^3+^-ZIF-90 (at identical concentrations presented in the hybrid composite) is presented in curve (ii). A 5-fold enhancement of the three-catalyst cascade in the frameworks, as compared to the diffusive cascaded process of the separated constituents, is observed. The effective operation of the cascade by the MOFs is attributed to the confined environment provided by the porous particles. The spatial proximity between the catalysts provides effective substrate/product transport tunnelling pathways intercommunicating the catalysts, thereby overcoming the diffusive barrier present in solution, resulting in the effective operation of the three-catalyst cascade in the MOF particles. As shown in Fig. 3D, panel II depicts the time-dependent concentration changes of TMB˙^+^ generated by the cascaded β-Gal/GOx/Fe^3+^-ZIF-90 in the presence of different concentrations of lactose. As the concentration of lactose increases, the rates of the generation of TMB˙^+^ are enhanced. As shown in Fig. 3D, panel III presents the rates of TMB˙^+^ formation as a function of lactose concentration. A saturation curve is observed demonstrating a *V*_max_ = 0.40 μM min^−1^ for the catalytic cascade operated by the composite β-Gal/GOx-loaded Fe^3+^-ZIF-90 MOFs.

**Fig. 4:**
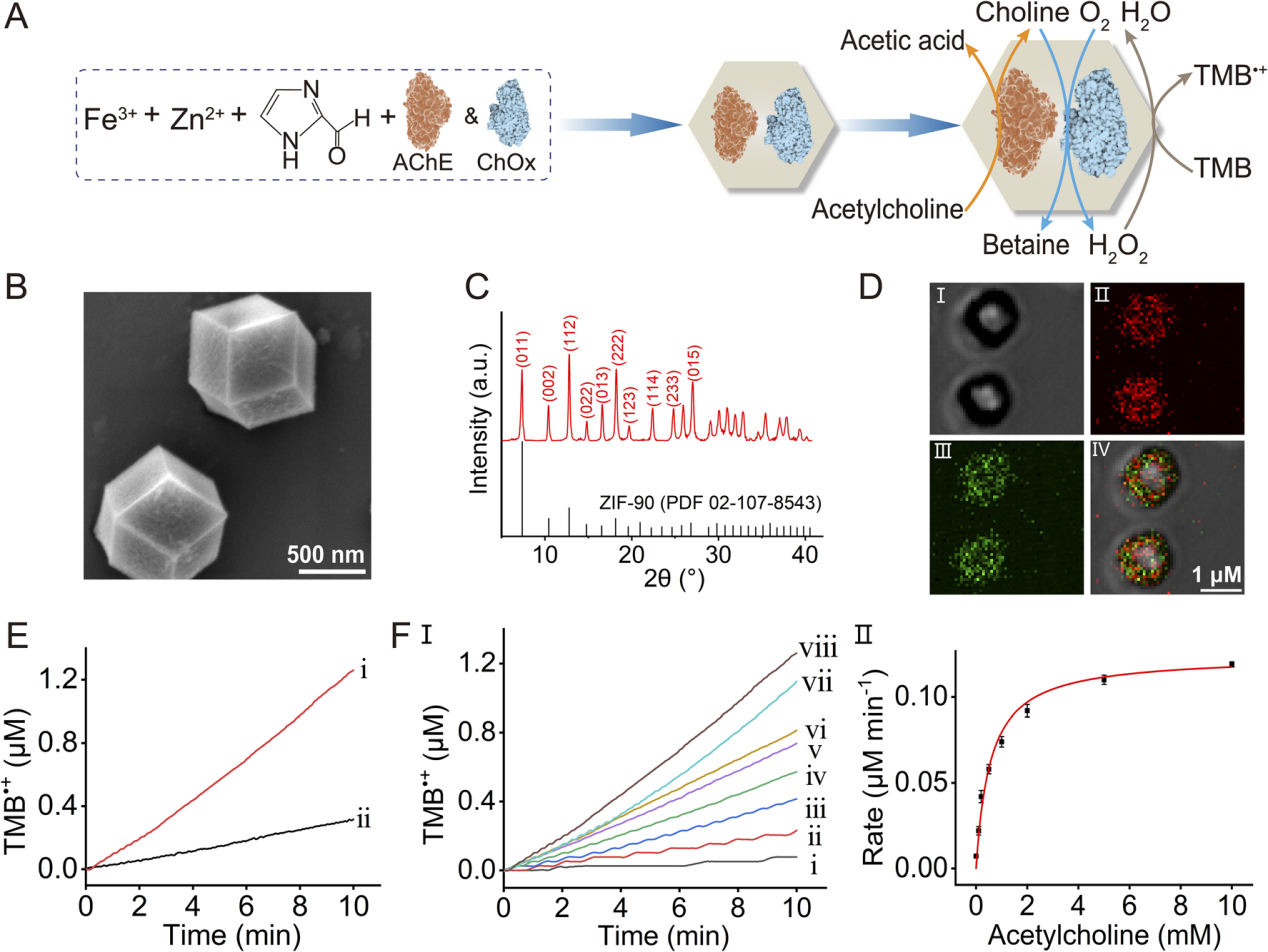
(A) Schematic assembly of the two enzymes (AChE and ChOx) in the porous Fe^3+^-ZIF-90 particles and their use as a three-catalyst cascade driving the AChE-catalyzed hydrolysis of acetylcholine, the subsequent aerobic oxidation of choline and the cascaded Fe^3+^-ZIF-90-catalyzed oxidation of TMB by H_2_O_2_ generated by the biocatalytic cascade. (B) Panel I: SEM image of the AChE/ChOx-loaded Fe^3+^-ZIF-90 MOFs. (C) XRD spectrum of the AChE/ChOx-loaded Fe^3+^-ZIF-90 particles: top—experimental results; bottom—spectral bands of ZIF-90 from the PDF database. (D) Bright field and fluorescence confocal microscopy images corresponding to the Atto 565-labeled AChE/FITC-labeled ChOx loaded in the Fe^3+^-ZIF-90: panel I—bright field image; panel II—imaging the Atto 565-labeled AChE (*λ*_ex_ = 561 nm, *λ*_em_ = 585 nm); panel III—imaging FITC-labeled ChOx (*λ*_ex_ = 488 nm, *λ*_em_ = 525 nm); panel IV—overlapped image. (E) Time-dependent concentration changes of TMB˙^+^ upon operating the acetylcholine-driving three-catalyst cascade using: (i) the AChE/ChOx-loaded Fe^3+^-ZIF-90 MOFs as an integrated assembly; (ii) the separated AChE, ChOx and Fe^3+^-ZIF-90 constituents in the homogeneous aqueous phase at concentrations identical to those in (i). (F) Panel I—time-dependent concentration changes of TMB˙^+^ upon operating the AChE/ChOx/Fe^3+^-ZIF-90 cascade in the presence of variable concentrations of acetylcholine: (i) 0 mM; (ii) 0.1 mM; (iii) 0.2 mM; (iv) 0.5 mM; (v) 1 mM; (vi) 2 mM; (vii) 5 mM; (viii) 10 mM. Panel II—rates of TMB˙^+^ formation by the AChE/ChOx/Fe^3+^-ZIF-90 system in the presence of variable concentrations of acetylcholine.

In the next step, we searched for an application of operating a biocatalytic cascade in the Fe^3+^-ZIF-90 frameworks that utilizes the advantages of operating a bienzyme/nanozyme cascaded reaction in the confined Fe^3+^-ZIF-90 microenvironment. This is exemplified in Fig. 4 with the engineering of bienzyme-loaded Fe^3+^-ZIF-90 frameworks composed of acetylcholine esterase, AChE, and choline oxidase, ChOx, with the vision that operation of the three-catalyst cascade in the frameworks could be implemented for sensing applications. Acetylcholine is a major neurotransmitter that signals cholinergic neurons. It is rapidly hydrolyzed by the serine protease, AChE, and any perturbation of binding acetylcholine to the neuronal synaptic membrane or inhibition of AChE can lead to neurological disorders, such as movement perturbation, paralysis and cognition.^65^ Perturbations of the levels of acetylcholine were reported to be associated with Alzheimer's and Parkinson's diseases as well as paranoid schizophrenia.^66–68^ Moreover, blockage of the cholinergic neurons or inhibiting AChE by organophosphorus pesticides or chemical warfare toxins may lead to severe neuronal disorders. Indeed, monitoring the levels of acetylcholine in plasma or the activity of AChE in the presence of potential inhibitors is an important analytical issue. Different analytical tools, such as mass spectrometry,^69^ high-performance liquid chromatography^70^ and radiolabeling,^71^ were employed to quantitatively detect acetylcholine. Also, diverse optical, electrochemical and enzymatic biosensing platforms following AChE activity were developed.^72,73^ For example, optical sensing platforms include surface plasma resonance,^74^ chemiluminescence,^75^ colorimetric assays^76^ and fluorescence sensing assays using semiconductor quantum dots.^77^ Also, electrochemical sensing platforms including amperometric biosensor devices,^78^ field-effect^79,80^ transistors and photoelectrochemical sensors^81^ following AChE activities and AChE inhibitors were reported. However, the present sensing platforms suffer from limited sensitivity, lack of desirable integration and circuit complexity. Thus, the integration of the sensing platform as a cascaded circuit in the MOF systems could provide a sensitive sensing platform due to the integrated nature of the system in the confined microenvironment. Fig. 4A depicts schematically the configuration of the three-catalyst cascaded frameworks. The two enzymes, AChE and ChOx, are integrated into the Fe^3+^-ZIF-90 MOFs. The AChE catalyzes the hydrolysis of acetylcholine, yielding acetic acid and choline as products. The aerobic oxidation of choline catalyzed by ChOx generates betaine and H_2_O_2_. The subsequent Fe^3+^-ZIF-90-catalyzed H_2_O_2_ oxidation of TMB to colored TMB˙^+^, provides an optical means to follow the catalytic cascade. The inhibition of AChE is, then, anticipated to affect the catalytic cascade. Evidently, appropriate engineering of the three-catalyst component, the structural and functional characterization of the framework and its response to inhibiting agents are essential for the assembly of the sensing platform. Accordingly, a stepwise process to assemble the sensor was adopted. In the first step, the single enzyme, ChOx, was integrated into the Fe^3+^-ZIF-90 MOFs. The ChOx-loaded particles and the accompanying aerobic oxidation of choline were characterized. Fig. S11† summarizes the structural and functional features of the ChOx-loaded Fe^3+^-ZIF-90 system. Evidently, the morphology and crystallinity of the Fe^3+^-ZIF-90 frameworks are preserved upon incorporation of ChOx. Also, the ChOx integrated into the composite demonstrates choline concentration-dependent activity. In the next step, the two enzymes, AChE and ChOx, were integrated into the Fe^3+^-ZIF-90 matrices, as shown in Fig. 4A. The morphology and crystallinity of the bienzyme-loaded particles were similar to those of the bare unloaded particles, as shown in Fig. 4B and C. The loading of the Fe^3+^-ZIF-90 particles by the two enzymes and the quantitative loading degree were evaluated by labeling the two enzymes with two different fluorophores and by complementary application of fluorescence confocal microscopy. ChOx was labeled with FITC (*λ*_ex_ = 488 nm, *λ*_em_ = 525 nm) and AChE was modified with Atto 565 (*λ*_ex_ = 561 nm, *λ*_em_ = 585 nm). Fig. 4D depicts the bright field and fluorescence confocal microscopy images using the different Atto 565 and FITC fluorescence channels and the merged image, panels I–IV. Evidently, the two enzymes were encapsulated in the Fe^3+^-ZIF-90 particles. For quantitative evaluation of the loading degree by the two fluorophore-modified enzymes in the frameworks, the residual fluorescence intensities of FITC–ChOx and Atto 565–AChE in the bulk solution after encapsulation were recorded. Knowing the original concentrations of the enzymes prior encapsulation and using appropriate calibration curves relating the concentrations of the fluorophore-modified enzymes and their associated fluorescence intensities, the contents of the Atto 565–AChE and FITC–ChOx in the MOF particles were evaluated to be 81.5 μg mg^−1^ particles and 86.8 μg mg^−1^ particles, respectively (see Fig. S12 and S13†). Moreover, the loading of the two enzymes in the Fe^3+^-ZIF-90 particles was confirmed by operating the three-catalyst cascade, as shown in Fig. 4E, curve (i), depicting the time-dependent concentration changes upon formation of TMB˙^+^ by the system. The control experiment, excluding ChOx and AChE from the particles, prohibited the formation of TMB˙^+^, confirming that the bienzyme-activated cascade is, indeed, operating. Furthermore, Fig. 4E, curve (ii) depicts the temporal formation of TMB˙^+^ in a diffusional mixture composed of separated AChE, ChOx and Fe^3+^-ZIF-90 particles (at identical concentrations to those of the integrated AChE/ChOx/Fe^3+^-ZIF-90 particles). The cascaded generation of TMB˙^+^ by the integrated assembly is *ca.* 5-fold enhanced as compared to the diffusional catalyst mixture, demonstrating the advantages of operating the catalytic cascade in the confined environment of the particles. Fig. 4F presents the time-dependent concentration changes of TMB˙^+^ generated by the integrated three-catalyst reactor in the presence of variable concentrations of acetylcholine. As the concentration of acetylcholine increases, the catalytic cascade is enhanced (Fig. 4F, panel I). Using the extinction coefficient of TMB˙^+^ (*ε* = 39 000 M^−1^ cm^−1^), the rates of TMB˙^+^ formation at different acetylcholine concentrations were evaluated (Fig. 4F, panel II), demonstrating the capacity of the AChE/ChOx/Fe^3+^-ZIF-90 to act as an optical platform for sensing acetylcholine. The detection limit for analyzing acetylcholine was determined to be 78 μM (for comparison of the acetylcholine sensing capacity by the AChE/ChOx/Fe^3+^-ZIF-90 framework, as compared to other sensing platforms, see Table S1†).

**Fig. 5:**
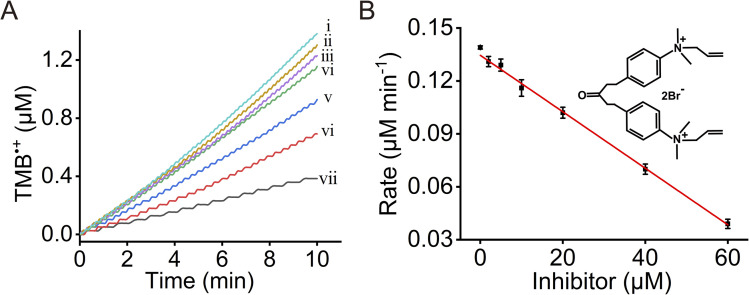
(A) Time-dependent concentration changes of TMB˙^+^ generated by the AChE/ChOx/Fe^3+^-ZIF-90 particles in the presence of acetylcholine, 10 mM, and variable concentrations of the 1,5-bis(4-allyldimethylammoniumphenyl)pentane-3-one dibromide (BW284C51) inhibitor: (i) 0 μM; (ii) 2 μM; (iii) 5 μM; (iv) 10 μM; (v) 20 μM; (vi) 40 μM; (vii) 60 μM. (B) Rates of TMB˙^+^ formation by the AChE/ChOx/Fe^3+^-ZIF-90 MOFs in the presence of variable concentrations of the BW284C51 inhibitor.

The results demonstrate that the integrated AChE/ChOx/Fe^3+^-ZIF-90 system provides an effective sensing platform for probing acetylcholine quantitatively. Furthermore, we find that the sensing capacity of the integrated framework is affected by the concentration of AChE in the frameworks, as shown in Fig. S14.† This suggests that the sensing performance of the AChE/ChOx/Fe^3+^-ZIF-90 frameworks will be affected by AChE inhibiting agents (*e.g.*, chemical warfare agents). Accordingly, the effect of the 1,5-bis(4-allyldimethylammoniumphenyl)pentane-3-one dibromide (BW284C51) inhibitor,^82^ a well-established inhibitor of AChE and a model for the chemical warfare agent, on the sensing performance of the three-catalyst cascade was examined. Fig. 5A presents the time-dependent concentration changes of the generated TMB˙^+^. As the concentration of the BW284C51 inhibitor increases, the activity of the AChE/ChOx/Fe^3+^-ZIF-90 catalytic cascade decreases. Fig. 5B depicts the rates of TMB˙^+^ formation catalyzed by the AChE/ChOx/Fe^3+^-ZIF-90 sensing platform in the presence of different concentrations of the BW284C51 inhibitor. The inhibitor could be sensed by the catalytic cascade with a detection limit of 1.69 μM.

## Conclusions

The present study has introduced Fe^3+^-doped ZIF-90 particles as functional frameworks for the assembly of biocatalyst/nanozyme composites acting as nanoreactors for the operation of catalytic cascades. The important functional features of the nanoreactor were as follows: (i) the Fe^3+^-ZIF-90 particles revealed peroxidase-like activities. (ii) The porous structure of the particles and their synthesis in an aqueous environment at room temperature enabled the high-loading encapsulation of multienzyme systems in the particles without affecting their activities. (iii) Coupling of the enzyme cascades with the peroxidase-like activities of the frameworks enabled the operation of three-catalyst cascades. (iv) The advantages of operating the enzymes in the confined porous frameworks, overcoming diffusional barriers present in the mixture of separated constituents of the catalytic cascades, were demonstrated. These included enhanced catalytic cascades in the confined particles as compared to the analogous diffusional assemblies. (v) The operation of the AChE/ChOx/Fe^3+^-ZIF-90 three-catalyst cascade was applied to develop a sensing framework monitoring agents inhibiting AChE, thereby providing a detection platform for the identification of hazardous chemical warfare. The concept of encapsulation of enzymes into the metal ion-doped ZIF to yield catalytic nanozyme-based framework systems can be extended to many other enzymes and other metal-ion dopants of the particles. Moreover, the ZIF-90 includes carboxaldehyde functionalities that could provide anchoring sites for chemical functionalities such as aptamers or antibodies. Such hybrid systems are envisaged to provide superior systems for sensing platforms and therapeutic applications.

## Data availability

The data supporting this article have been included as part of the ESI.†

## Author contributions

I. Willner and X. Chen conceived and designed the experiments. J. Wang carried out the synthesis and catalytic activity test of the materials. Y. Qin, R. Carmieli and V. Gutkin contributed to the characterization studies. Z. Zhang and E. Pikarsky participated in the analysis of the results. I. Willner, J. Wang and X. Chen designed the figures and co-wrote the manuscript. All authors reviewed and approved the final manuscript.

## Conflicts of interest

There are no conflicts to declare.

## Supplementary Material

SC-016-D5SC01972A-s001
