# Supplementary material for: Enzyme-loaded Fe3+-doped ZIF-90 particles as catalytic bioreactor hybrids for operating catalytic cascades
Source: Chem Sci. 2025 May 1;16(22):9705–14. doi: 10.1039/d5sc01972a (PMC12044419; doi:10.1039/d5sc01972a)
Supplement: SC-016-D5SC01972A-s001 [file SC-016-D5SC01972A-s001.pdf]

Supplementary Information (SI) for Chemical Science.
This journal is © The Royal Society of Chemistry 2025

Supporting Information for

Enzyme-Loaded Fe^{3+}-Doped ZIF-90 Particles as Catalytic Bioreactor Hybrids Operating Catalytic Cascades

Jin Wang, [a,b] Yunlong Qin, [a] Raanan Carmieli, [c] Vitaly Gutkin, [d] Eli Pikarsky, [e] Zhen Zhang, *[b] Xinghua Chen, *[a] and Itamar Willner *[a]

[a.] *Institute of Chemistry, The Hebrew University of Jerusalem, Jerusalem 91904, Israel.*

[b.] *School of the Environment and Safety Engineering, Jiangsu University, Zhenjiang 212013, China.*

[c.] *Department of Chemical Research Support, Weizmann Institute of Science, Rehovot 76100, Israel.*

[d.] *The Center for Nanoscience and Nanotechnology, The Hebrew University of Jerusalem, Jerusalem 91904, Israel.*

[e.] *Faculty of Medicine, The Hebrew University of Jerusalem, Jerusalem 91120, Israel.*

Email: Itamar.Willner@mail.huji.ac.il; Xinghua.Chen@mail.huji.ac.il; zhangzhan@ujs.edu.cn

Table of Contents

Experimental Section P1

Abbreviations..P6

Fig. S1. XPS spectra of Fe^{3+}-ZIF-90 ....P7

Fig. S2. N_2 adsorption/desorption isotherm of Fe^{3+}-ZIF-90 ....P8

Fig. S3. NADH oxidation by H_2O_2 using Fe^{3+}-ZIF-90...P9

Fig. S4. Probing ROS intermediates by DPBF P10

Suggested mechanism for the peroxidase-like activity of Fe^{3+}-ZIF-90..................................P11

Fig. S5. Quantifying the loading of GOx in GOx-loaded Fe^{3+}-ZIF-90 by GOx/HRP assay..P12

Fig. S6. Confocal images of GOx-loaded Fe^{3+}-ZIF-90 ....P13

Fig. S7. Quantifying the loading of GOx in GOx-loaded Fe^{3+}-ZIF-90 by fluorescence method P14

Fig. S8. Probing the cascaded oxidation of TMB by GOx-loaded Fe^{3+}-ZIF-90 with various loading of GOx P15

Fig. S9. Quantifying the loading of GOx in β-Gal/GOx-loaded Fe^{3+}-ZIF-90........................P16

Fig. S10. Quantifying the loading of β-Gal in β-Gal/GOx-loaded Fe^{3+}-ZIF-90.....................P17

Fig. S11. Structural and functional features of ChOx-loaded Fe^{3+}-ZIF-90 ....P18

Fig. S12. Quantifying the loading of ChOx in AChE/ChOx-loaded Fe^{3+}-ZIF-90 ....P19

Fig. S13. Quantifying the loading of AChE in AChE/ChOx-loaded Fe^{3+}-ZIF-90 ....P20

Fig. S14. Probing the cascaded oxidation of TMB by AChE/ChOx-loaded Fe^{3+}-ZIF-90 with various loadings of AChE P21

Table S1. Comparison of acetylcholine detection limit of different methods P22

References P23

Experimental Section

(For a detailed list of abbreviations of the materials and techniques used in the study, see Supporting Information P6)

Materials. Imidazole-2-carboxaldehyde (2-ICA) was purchased from Alfa Aesar. Polyvinylpyrrolidone (PVP, MW: 40000), zinc nitrate hexahydrate ($Zn(NO_3)_2 \cdot 6H_2O$), iron (III) chloride ($FeCl_3$), glucose oxidase (GOx, 127 units mg^{-1}), β-galactosidase (β-Gal, 500 units mg^{-1}), choline oxidase (ChOx, 12 units mg^{-1}), acetylcholinesterase (AChE, 658 units mg^{-1}), glucose, β-lactose, acetylcholine, choline, 3,3',5,5'-Tetramethylbenzidine (TMB), 1,4-dihydronicotinamide adenine dinucleotide (NADH), hydrogen peroxide solution (30 % in H_2O), Atto 565 NHS ester, fluorescein isothiocyanate isomer I (FITC), dimethyl sulfoxide (DMSO), 5-tert-butoxycarbonyl-5-methyl-1-pyrroline-N-oxide (BMPO), 1,5-bis(4-allyldimethylammoniumphenyl)pentane-3-one dibromide (BW284C51), magnesium chloride ($MgCl_2$), sodium chloride (NaCl), disodium hydrogen phosphate (Na_2HPO_4), sodium dihydrogen phosphate (NaH_2PO_4), Trizma® hydrochloride and 2-(N-morpholino)ethanesulfonic acid (MES) were ordered from Sigma-Aldrich (USA). Ultrapure water (18.2 MΩ cm) used in all experiments was purified by a NANOpure Diamond (Barnstead Int., USA).

Characterizations. The UV-vis absorption spectra and kinetic measurements were performed on a UV-2450 UV-vis spectrophotometer (Shimadzu, Japan) using a quartz cuvette with the pathlength of 1 cm. The fluorescence spectra were acquired on a Cary Eclipse fluorometer (Agilent, USA). The scanning electron microscopy (SEM) images were taken by using an Extra-High Resolution Scanning Electron Microscope Magellan 400L (Thermo Fisher, USA). The powder X-ray diffraction (PXRD) spectra were recorded on an X-ray powder diffractometer D8 Advance with 2.2 kW Cu Kα X-ray source mounted into the TWIST-TUBE assembly (Bruker, USA). The X-ray photoelectron spectroscopy (XPS) spectra were collected using a Kratos AXIS Supra spectrometer with Al Kα monochromatic radiation X-ray source (1486.6 eV) as the excitation source (Kratos, UK). The metal content was measured by an Agilent 7500cx

inductively coupled plasma mass spectrometer (Agilent, USA). The confocal fluorescence microscopy images were obtained using an Olympus FV3000 confocal laser-scanning microscope (Olympus, Japan). The electron paramagnetic resonance (EPR) measurements were conducted on a Bruker ELEXSYS E500 spectrometer operating at X-band frequencies (9.5 GHz) and a Bruker ER4102ST resonator (Bruker, USA). Samples were loaded into Vitrocom quartz capillaries, CV1012-Q-100, with 1 mm inner diameter. For $TMB^{\bullet+}$ measurement, 20 mW microwave power, 0.1 Gauss modulation amplitude, 100 kHz modulation frequency, 40 s conversion time, and 50 G sweep range were used. The spectrum was simulated using a MATLAB program based on easyspin subroutines.

Synthesis of Fe^{3+}-ZIF-90. The Fe^{3+}-ZIF-90 was synthesized according to a previous report[1] with slight modification. Briefly, 38.4 mg (0.4 mmol) of 2-ICA and 40 mg of PVP were dissolved in a solution containing 1.8 ml of water and 0.2 ml of ethanol by heating and sonicating. Then, 1 ml of solution composed of $Zn(NO_3)_2 \cdot 6H_2O$ (29.95 mg, 0.1 mmol) and $FeCl_3$ (0.8 mg, 0.005 mmol) were added into the above mixture dropwise under stirring. After 1 hour, the Fe^{3+}-ZIF-90 was obtained by centrifugation (4000 rpm, 5 min) and washing three times with ultrapure water.

Synthesis of FITC-labeled GOx. 100 μL of GOx (1.6 mg in water) was mixed with 50 μL of FITC (1 mM in DMSO) and then added to 350 μL of phosphate buffer (10 mM, pH 9). The obtained mixture of GOx and FITC was incubated at 4 °C in the dark for 8 h, followed by washing with water using an Amicon filter (10 KDa MWCO) for three times (8000 rpm, 10 min) to get FITC-labeled GOx.

Preparation of Atto 565-labeled β-Gal. 100 μL of β-Gal (1 mg in water) was mixed with 50 μl of Atto 565 (0.1 mM in DMSO) and added into 350 μL of phosphate buffer (10 mM, pH 8). The mixture was incubated at 4 °C in the dark for 8 h and then washed with water using an Amicon filter (10 KDa MWCO) for three times (8000 rpm, 10 min) to obtain Atto 565-labeled β-Gal.

Synthesis of FITC-labeled ChOx. 100 μL of ChOx (0.83 mg in water), 50 μL of FITC (1 mM in DMSO) and 350 μL phosphate buffer (10 mM, pH 9) were mixed and

then incubated at 4 °C in the dark for 8 h. followed by washing with water using an Amicon filter (10 KDa MWCO) for three times (8000 rpm, 10 min) to get FITC-labeled ChOx.

Preparation of Atto 565-labeled AChE. 100 μL of AChE (0.7 mg in water), 50 μl of Atto 565 (0.1 mM in DMSO) and 350 μL phosphate buffer (10 mM, pH 9) were mixed. The obtained mixture was incubated at 4 °C in the dark for 8 h and then washed with water using an Amicon filter (10 KDa MWCO) for three times (8000 rpm, 10 min) to collect Atto 565-labeled AChE.

Preparation of enzyme-loaded Fe^{3+}-ZIF-90. The preparation of enzyme-loaded Fe^{3+}-ZIF-90 followed the synthesis of Fe^{3+}-ZIF-90 with modifications. In short, 38.4 mg of 2-ICA and 40 mg of PVP were fully dissolved in ethanol/water (1:9 in volume) solution by heating and sonicating. The mixture was then cooled down for 5 minutes to prevent enzyme inactivation. For the preparation of β-Gal/GOx-loaded Fe^{3+}-ZIF-90, 100 μL of solution containing 0.7 mg of β-Gal and 0.8 mg of GOx was added to the cooled mixture of 2-ICA and PVP. Similarly, for the preparation of AChE/ChOx-loaded Fe^{3+}-ZIF-90, 100 μL of solution containing of 0.8 mg of AChE and 0.83 mg of ChOx was used. After enzyme addition, 1 mL of solution consisting of $Zn(NO_3)_2 \cdot \bullet$ $6H_2O$ (29.95 mg, 0.1 mmol) and $FeCl_3$ (0.8 mg, 0.005 mmol) were introduced to initiate the formation of Fe^{3+}-ZIF-90 encapsulating enzymes. The bienzyme-loaded Fe^{3+}-ZIF-90 was obtained after stirring for 1 h and then purifying by centrifugation at 4000 rpm for 5 minutes and washing three times with water. The single enzyme-loaded Fe^{3+}-ZIF-90 was prepared following a similar procedure but with GOx or ChOx solution at the same concentration with bienzyme-loaded MOFs. For GOx-loaded Fe^{3+}-ZIF-90 and AChE/ChOx-loaded Fe^{3+}-ZIF-90, various amount of GOx (1.2 mg and 1.6 mg) or AChE (0.4 mg, 0.6 mg and 1 mg) were also used to prepare enzyme-loaded Fe^{3+}-ZIF-90 with different loading following the same procedure, which were only used in the experiments to reveal the effect of enzyme loading on the cascaded catalytic activity.

Catalytic H_2O_2 Oxidation of TMB to $TMB^{\bullet+}$ by Fe^{3+}-ZIF-90. Briefly, Tris-HCl (pH 7.4), TMB and Fe^{3+}-ZIF-90 were mixed in sequence with the final concentration

of 10 mM, 2 mM and 100 μg mL^{-1}, respectively. Then, H_2O_2 with various concentrations (0, 0.1, 0.2, 0.5, 1, 2, 5 and 10 mM) was added. Kinetic mode with the time interval of 1 s was conducted immediately to monitor the absorbance changes of the generated $TMB^{•+}$ at 652 nm by a UV-2450 spectrophotometer at room temperature (RT). The concentration of the generated $TMB^{•+}$ was calculated by the Beer's law with $\varepsilon_{TMB•+, 652\ nm}$ = 39000 M^{-1} cm^{-1} and distance of 1 cm. The initial rates in the presence of different concentrations of H_2O_2 were calculated by the slope of the kinetic curves and correlated to the concentrations of H_2O_2 to get V_{max} of the catalyzed reaction by fitting to the Michaelis-Menten equation.

Catalytic H_2O_2 Oxidation of NADH to NAD^+ by Fe^{3+}-ZIF-90. Briefly, MES (pH 5.5), NADH, H_2O_2 and Fe^{3+}-ZIF-90 were sequentially mixed to the final concentration of 10 mM, 250 μM, 10 mM and 100 μg mL^{-1}, respectively, Repeat-scan mode with the time interval of 10 min was then started to record the time-dependent absorbance changes using a UV-2450 spectrophotometer at RT.

Catalytic oxidation of TMB to $TMB^{•+}$ by enzyme-loaded Fe^{3+}-ZIF-90. For β-Gal/GOx/Fe^{3+}-ZIF-90 system, Tris-HCl (pH 7.4), β-lactose, TMB and β-Gal/GOx/Fe^{3+}-ZIF-90 were sequentially mixed to the final concentration of 10 mM, 10 mM, 2 mM and 100 μg mL^{-1}, respectively, followed by the same measurement procedure of Fe^{3+}-ZIF-90-catalyzed oxidation of TMB by H_2O_2. The control experiment with separated β-Gal, GOx and Fe^{3+}-ZIF-90 constituents was conducted under the same condition of the integrated β-Gal/GOx/Fe^{3+}-ZIF-90 system. β-lactose at various concentrations (0, 0.1, 0.2, 0.5, 1, 2, 5 mM) were also used to drive the cascaded oxidation of TMB in the presence of integrated β-Gal/GOx/Fe^{3+}-ZIF-90 following the same procedure. For GOx/Fe^{3+}-ZIF-90, ChOx/Fe^{3+}-ZIF-90 and AChE/ChOx/Fe^{3+}-ZIF-90, the same procedure was conducted but using glucose, choline and acetylcholine to drive the bioenzyme/nanozyme cascades, respectively.

Inhibition effect of BW284C51 on the performance of AChE/ChOx/Fe^{3+}-ZIF-90. Briefly, various concentrations of BW284C51 (0, 2, 5, 10, 20, 40 and 60 μM) were mixed with Tris-HCl (10 mM, pH 7.4), acetylcholine (10 mM), TMB (2 mM) and

AChE/ChOx/Fe^{3+}-ZIF-90 (100 μg mL^{-1}) followed by the same measurement procedure of AChE/ChOx/Fe^{3+}-ZIF-90 system without inhibitor.

Identification of reactive oxygen species (ROS) and $TMB^{•+}$. ROS generated upon operating Fe^{3+}-ZIF-90-catalyzed H_2O_2 oxidation of TMB were identified by the EPR spin trapping technique using BMPO as the trapping agent. Specifically, Tris-HCl (pH 7.4), BMPO, H_2O_2 and Fe^{3+}-ZIF-90 were mixed in sequence to the final concentrations of 10 mM, 10 mM, 10 mM and 100 μg mL^{-1}, respectively, and then loaded into the capillary to start the measurement on a ELEXSYS E500 spectrometer at RT. The formation of •OH radicals and their involvement in the oxidation of TMB were further confirmed by adding 10% DMSO or 10 mM TMB, following the previously described procedure. To identify the formation of $TMB^{•+}$, Tris-HCl (pH 7.4), TMB, H_2O_2 and Fe^{3+}-ZIF-90 were mixed in sequence to the final concentrations of 10 mM, 2 mM, 10 mM and 100 μg mL^{-1}, respectively, and then loaded into the capillary to start the measurement on a ELEXSYS E500 spectrometer with the parameters described in the characterization section at RT.

The ROS were also studied by its quench effect on the absorbance of DPBF. Briefly, Tris-HCl (pH 7.4), DPBF, H_2O_2 and Fe^{3+}-ZIF-90 were sequentially mixed to the final concentration of 10 mM, 30 μM, 10 mM and 100 μg mL^{-1}, respectively. Repeat-scan mode with the time interval of 5 min was then started to record the time-dependent absorbance changes using a UV-2450 spectrophotometer at RT.

Abbreviations

MOFs: metal-organic frameworks

ZIF: zeolitic imidazolate framework

2-ICA: imidazole-2-carboxaldehyde

PVP: polyvinylpyrrolidone

MES: 2-(N-morpholino)ethanesulfonic acid

DMSO: dimethyl sulfoxide

FITC: fluorescein isothiocyanate isomer I

GOx: glucose oxidase

β-Gal: β-galactosidase

ChOx: choline oxidase

AChE: acetylcholine esterase

NADH: 1,4-dihydronicotinamide adenine dinucleotide

TMB: 3,3',5,5'-tetramethylbenzidine

DPBF: 1,3-diphenylisobenzofuran

BMPO: 5-tert-butoxycarbonyl-5-methyl-1-pyrroline-N-oxide

BW284C51: 1,5-bis(4-allyldimethylammoniumphenyl)pentane-3-one dibromide

ROS: reactive oxygen species

•OH: hydroxyl radicals

SEM: scanning electron microscopy

PXRD: powder X-ray diffraction

XPS: X-ray photoelectron spectroscopy

ICP-MS: inductively coupled plasma mass spectrometry

BET: Brunauer-Emmett-Teller

EPR: electron paramagnetic resonance

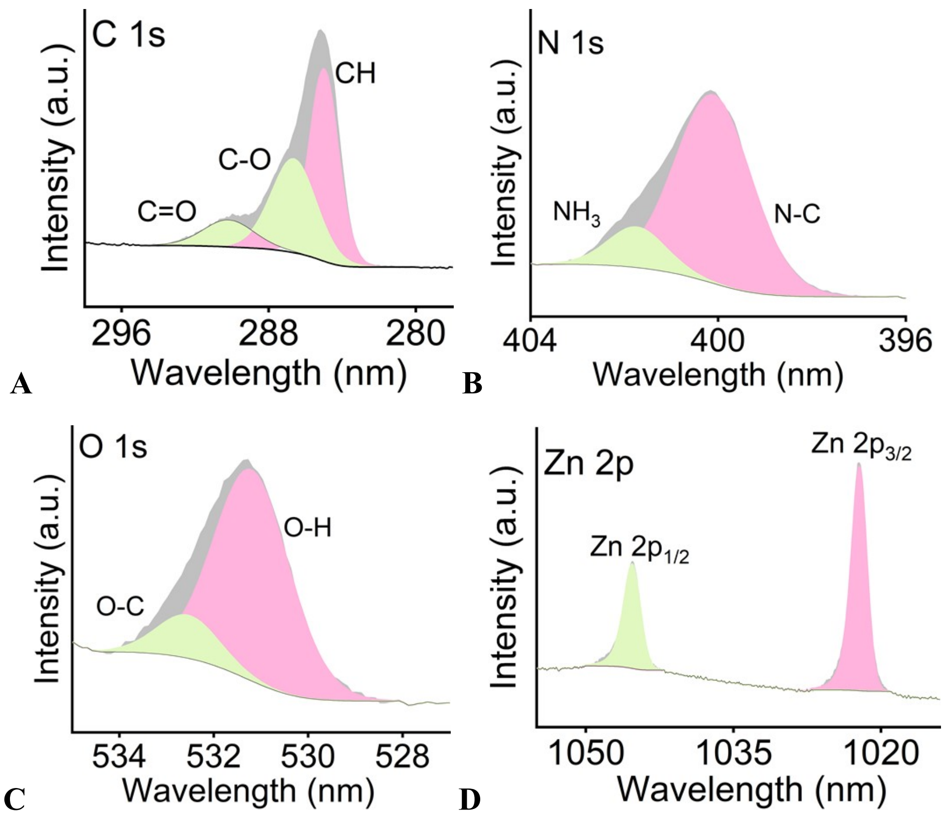

A B C D

Fig. S1. Deconvoluted C 1s (**A**), N 1s (**B**), O 1s (**C**) and Zn 2p (**D**) XPS spectra of the Fe^{3+}-ZIF-90 particles.

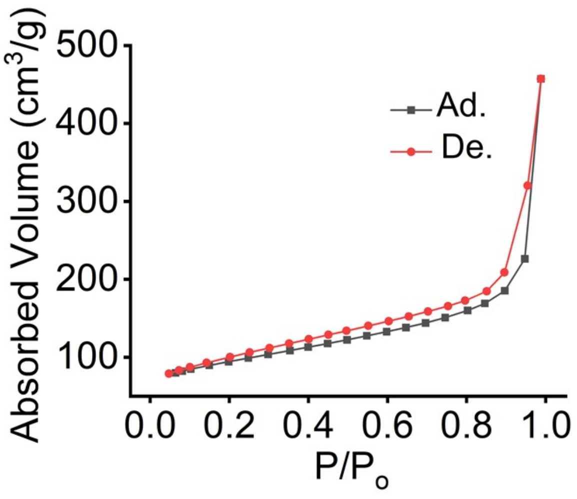

Fig. S2. N_2 adsorption/desorption isotherm of Fe^{3+}-ZIF-90 particles.

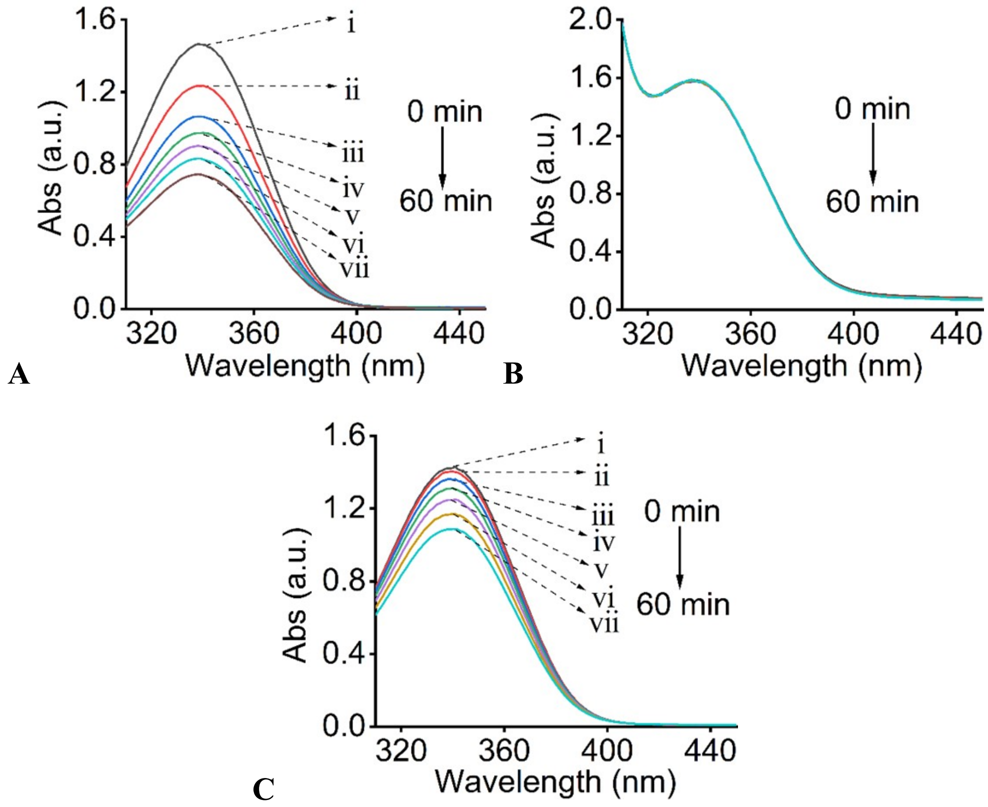

Fig. S3. Time-dependent absorbance changes of NADH (250 μM): **(A)** in the presence of H_2O_2, 10 mM, and Fe^{3+}-ZIF-90 particles, 100 μg mL^{-1}; **(B)** in the presence of Fe^{3+}-ZIF-90 particles, 100 μg mL^{-1}, and in the absence of H_2O_2; **(C)** in the presence of H_2O_2, 10 mM, and in the absence of Fe^{3+}-ZIF-90 MOFs. (**i**) 0 min; (**ii**) 10 min; (**iii**) 20 min; (**iv**) 30 min; (**v**) 40 min; (**vi**) 50 min; (**vii**) 60 min.

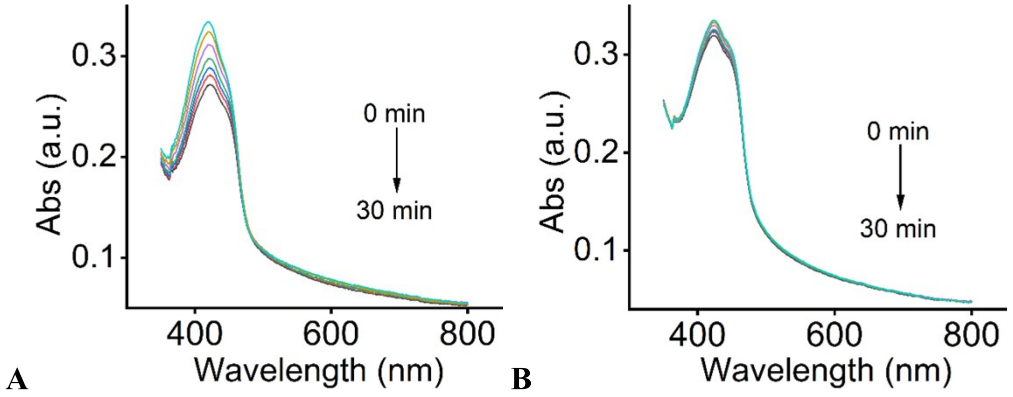

Fig. S4. Time-dependent absorbance changes of DPBF, 60 µM, as a result of ROS formation: **(A)** in the presence of H_2O_2, 10 mM, and Fe^{3+}-ZIF-90, 100 µg mL^{-1}; **(B)** in the presence of Fe^{3+}-ZIF-90 particles, 100 µg mL^{-1}, and in the absence of H_2O_2.

Mechanistic path corresponding to the Fe^{3+}-ZIF-90 peroxidase-like activity for generation of •OH.

Following previous reports,[2, 3] the suggested mechanistic pathway corresponding to the Fe^{3+}-ZIF-90-catalyzed transformation of H_2O_2 into •OH follows the steps outlined in eq. (1) - eq. (3):

(1) $Fe^{3+} + H_2O_2 \longrightarrow Fe^{2+} + O_2^{\bullet +} + 2H^+$

(2) $O_2^{\bullet +} + H^+ \xrightarrow{\text{disproportionation}} 1/2H_2O_2 + 1/2O_2$

(3) $Fe^{2+} + H_2O_2 + H^+ \longrightarrow Fe^{3+} + \bullet OH + H_2O$

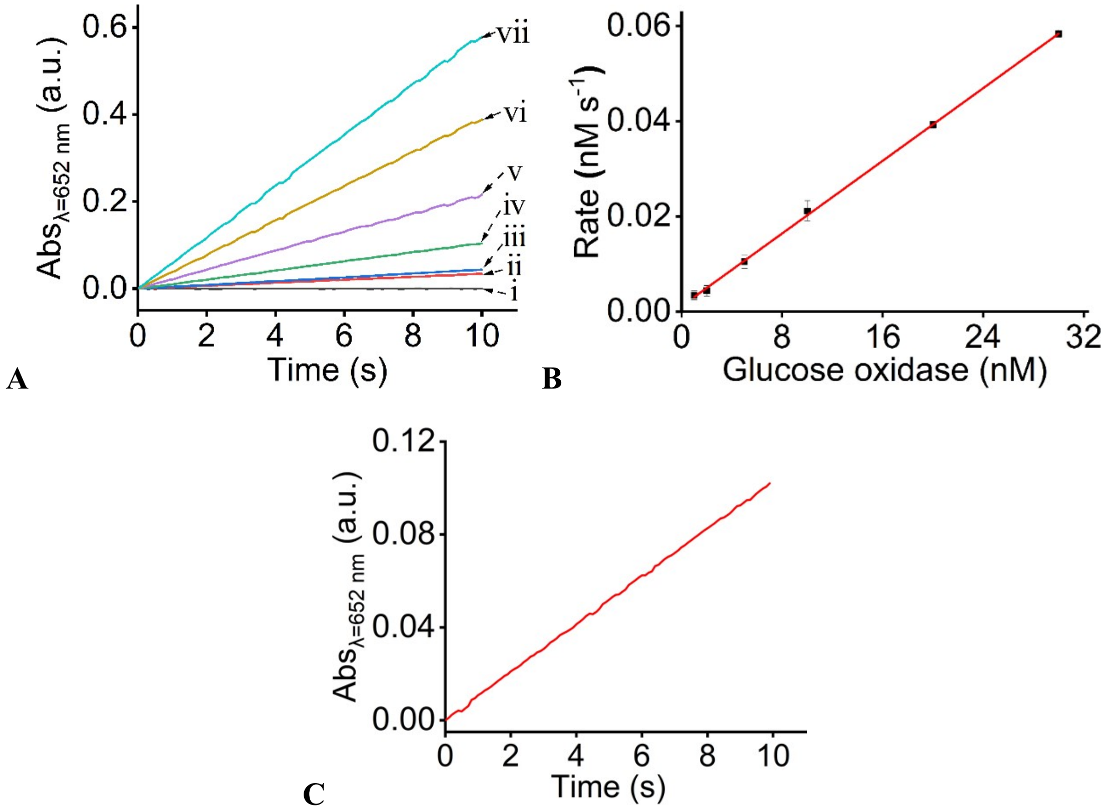

Fig. S5. (A) Time-dependent absorbance changes of $TMB^{\bullet+}$ formation in the presence of 2 mM TMB, 2 μM HRP, 1 mM glucose and different concentrations of glucose oxidase: (**i**) 0 nM; (**ii**) 1 nM; (**iii**) 2 nM; (**iv**) 5 nM; (**v**) 10 nM; (**vi**) 20 nM; (**vii**) 30 nM. (B) Rates of $TMB^{\bullet+}$ formation by the GOx/HRP system as a function of glucose oxidase concentrations. (C) Time-dependent absorbance changes of $TMB^{\bullet+}$ formation in the presence of 2 mM TMB, 2 μM HRP, 1 mM glucose and supernatant after formation of GOx-loaded Fe^{3+}-ZIF-90 MOFs.

A certain amount of GOx was used to prepare GOx-loaded Fe^{3+}-ZIF-90 MOFs. After formation of enzyme-loaded MOFs, centrifugation was conducted to obtain GOx-loaded ZIF-90 MOFs. Unloaded GOx remained in the supernatant are supposed to retain the catalytic activity of GOx. Thus, a calibration curve was plotted to establish the relationship between the rate of TMB oxidation and glucose oxidase concentration with the HRP/GOx assay. The oxidation of TMB driven by the GOx remained in the supernatant was then measured at the identical condition to those of HRP/GOx assay to calculate the rate of TMB oxidation, followed by applying the standard curve to calculate the concentration of unloaded GOx. Knowing the original concentration of GOx, the concentrations of the GOx encapsulated in the Fe^{3+}-ZIF-90 were evaluated to be 97.4 μg mL^{-1}.

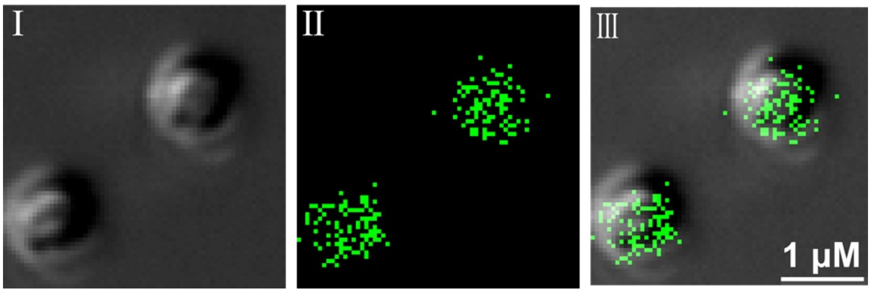

Fig. S6. Bright field and fluorescence confocal microscopy image corresponding to FITC-labeled GOx loaded in the Fe^{3+}-ZIF-90: **Panel I**-bright field; **Panel II**-imaging FITC-labeled GOx (λ_{ex}=488 nm, λ_{em}=525 nm); **Panel III**-overlapped image.

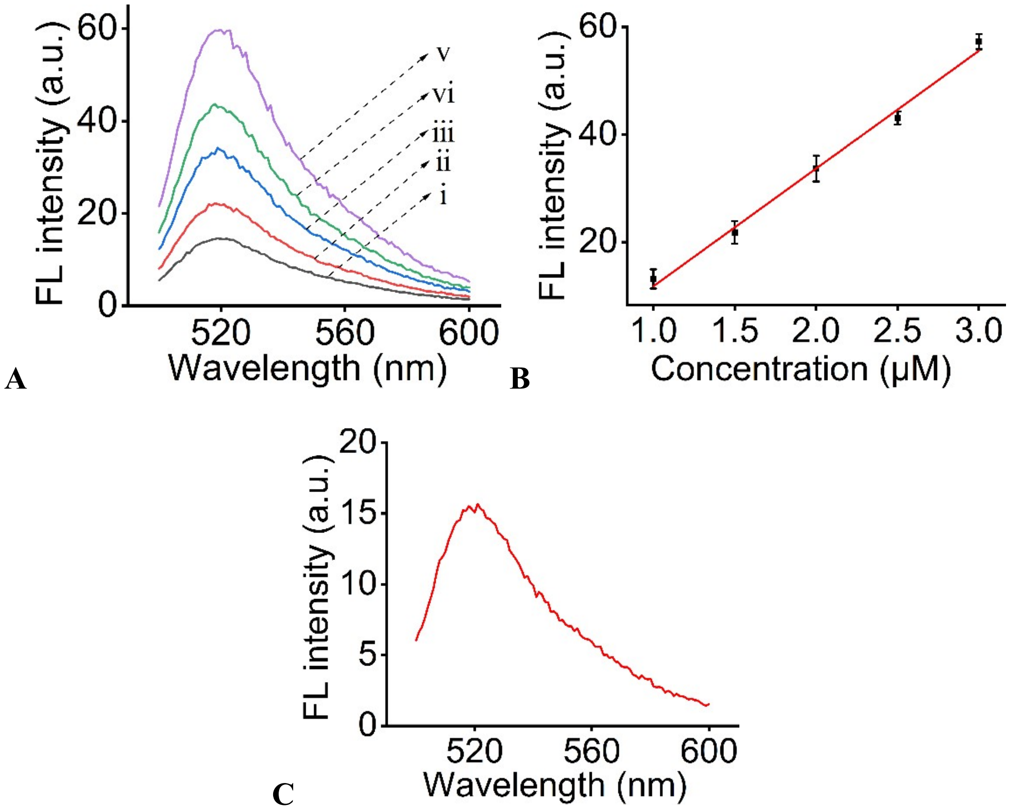

Fig. S7. (A) Fluorescence spectra of FITC-labeled GOx at different concentrations: (**i**) 1 μM; (**ii**) 1.5 μM; (**iii**) 2 μM; (**iv**) 2.5 μM; (**v**) 3 μM. (λ_{ex}=488 nm). **(B)** Linear relationship between the fluorescence intensity of FITC-labeled GOx at 520 nm and the concentration of FITC-labeled GOx. **(C)** Fluorescence spectra of unloaded FITC-labeled GOx in the supernatant after formation of FITC-labeled GOx-loaded Fe^{3+}-ZIF-90 MOFs (λ_{ex}=488 nm).

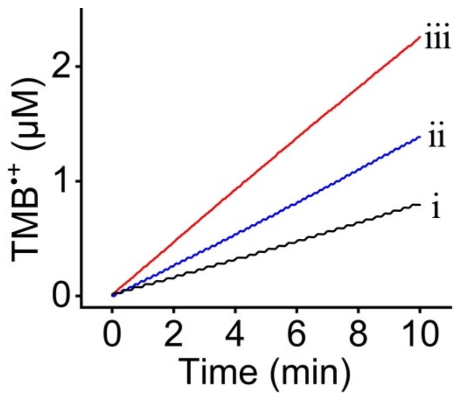

Fig. S8. Time-dependent concentration changes of $TMB^{\bullet+}$ upon glucose-driving cascade in the presence of GOx-loaded Fe^{3+}-ZIF-90 particles with various loading amount of GOx: (**i**) 50.8 μg mg^{-1}; (**ii**) 75.2 μg mg^{-1}; (**iii**) 97.4 μg mg^{-1}. In all experiment, 2 mM of TMB, 10 mM of glucose and 100 μg mg^{-1} GOx-loaded Fe^{3+}-ZIF-90 particles were used.

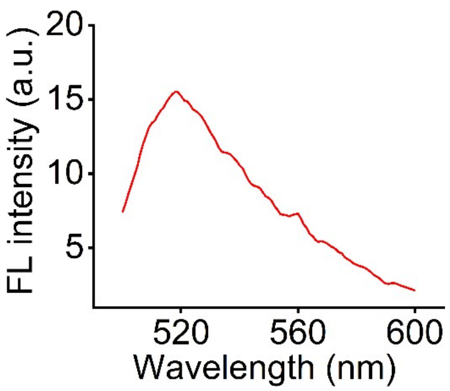

Fig. S9. Fluorescence spectra of unloaded FITC-labeled GOx in the supernatant after formation of 565 labeled β-Gal/FITC-labeled GOx-loaded Fe^{3+}-ZIF-90 MOFs (λ_{ex}=488 nm).

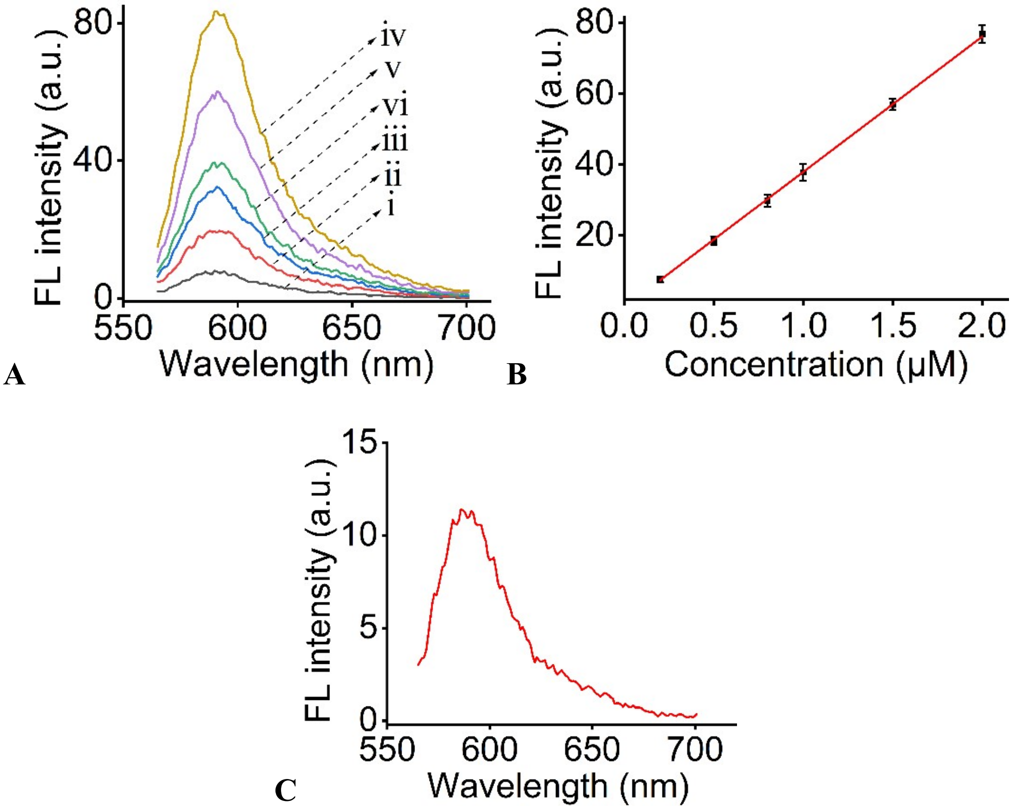

Fig. S10. (A) Fluorescence spectra of Atto 565 labeled β-Gal at different concentrations: (**i**) 0.2 μM; (**ii**) 0.5 μM; (**iii**) 0.8 μM; (**iv**) 1 μM; (**v**) 1.5 μM; (**iv**) 2 μM (λ_{ex}=561 nm). **(B)** Linear relationship between the fluorescence intensity of 565 labeled β-Gal at 585 nm and the concentration of Atto 565 labeled β-Gal. (**C**) Fluorescence spectra of unloaded Atto 565-labeled β-Gal in the supernatant after formation of β-Gal/GOx-loaded Fe^{3+}-ZIF-90 MOFs.

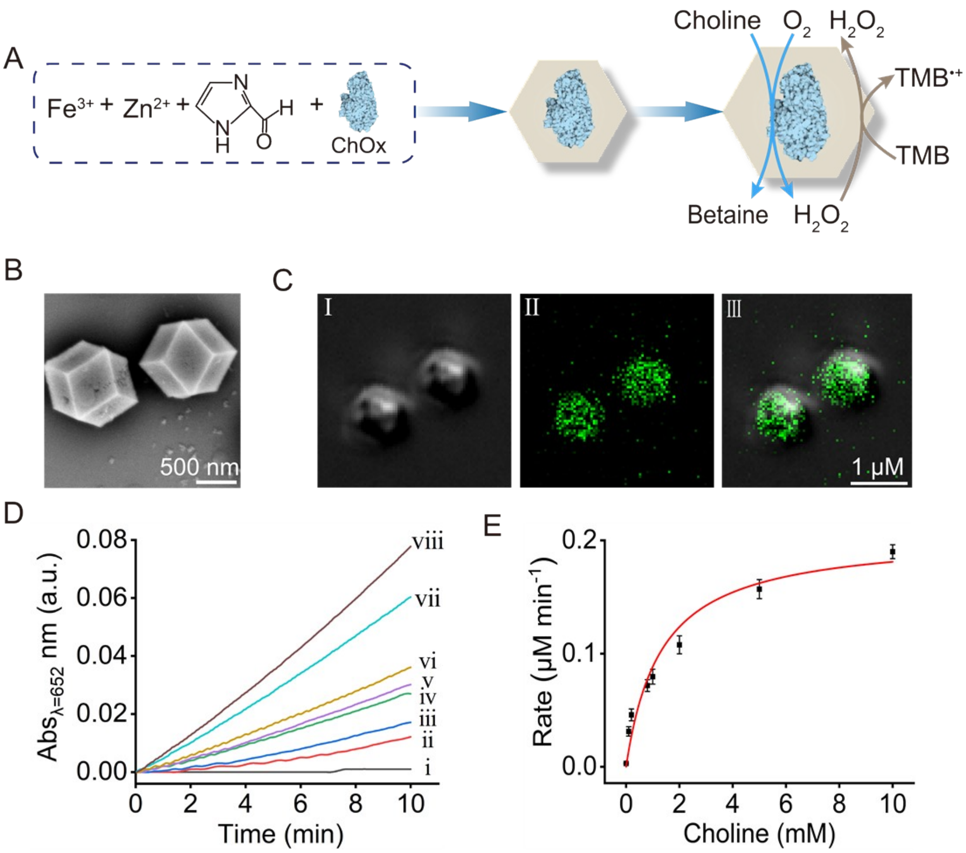

Fig. S11. (A) Schematic synthesis of ChOx-loaded Fe^{3+}-ZIF-90 particles and their use towards the cascaded ChOx-catalyzed aerobic oxidation of choline followed by the Fe^{3+}-ZIF-90-catalyzed oxidation of TMB to $TMB^{\bullet+}$ by the generated H_2O_2. **(B)** SEM image of ChOx-loaded Fe^{3+}-ZIF-90 particles. **(C)** Bright field and fluorescence confocal microscopy image corresponding to FITC-labeled ChOx loaded in the ChOx-loade Fe^{3+}-ZIF-90: **Panel I**-bright field; **Panel II**-imaging FITC-labeled ChOx (λ_{ex}=488 nm, λ_{em}=525 nm); **Panel III**-overlapped image. **(D)** Time-dependent absorbance changes of $TMB^{\bullet+}$ upon operating the ChOx-loaded Fe^{3+}-ZIF-90 framework, 100 μg mL^{-1}, TMB, 2 mM, in the presence of variable concentrations of choline: (**i**) 0 mM; (**ii**) 0.1 mM; (**iii**) 0.2 mM; (**iv**) 0.5 mM; (**v**) 1 mM; (**vi**) 2 mM; (**vii**) 5 mM; (**viii**) 10 mM. **(E)** Rates of TMB oxidation to $TMB^{\bullet+}$ by the ChOx-loaded Fe^{3+}-ZIF-90/TMB system as a function of choline concentrations.

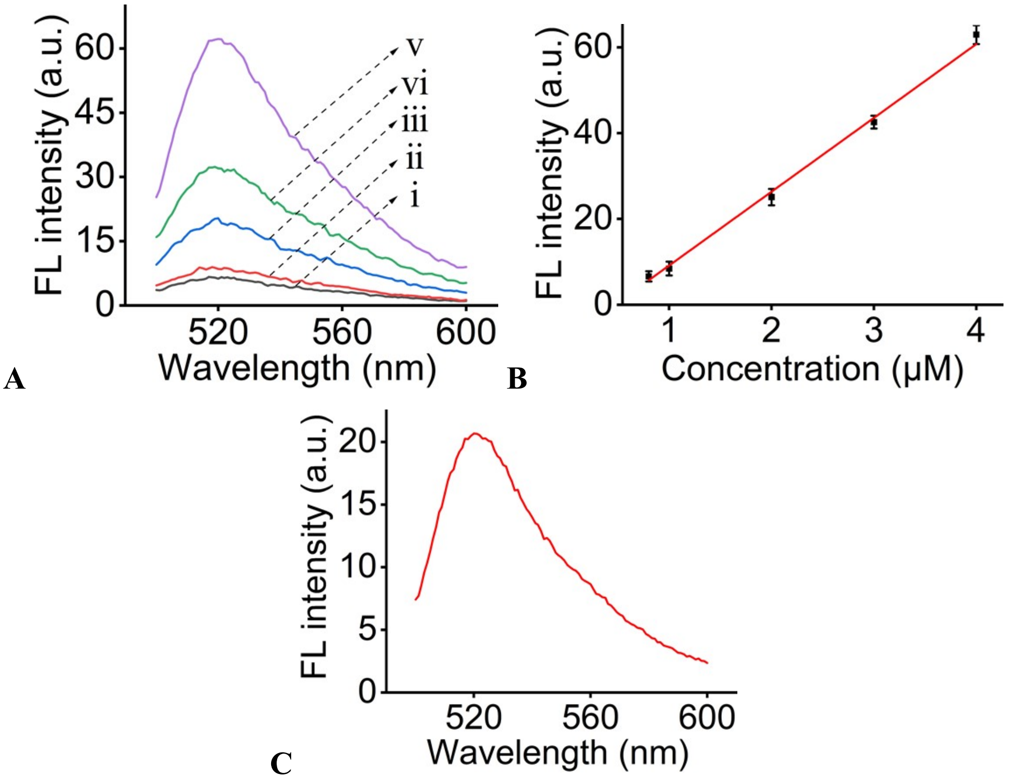

Fig. S12. (A) Fluorescence spectra of FITC-labeled ChOx at different concentrations: (**i**) 0.8 μM; (**ii**) 1 μM; (**iii**) 2 μM; (**iv**) 3 μM; (**v**) 4 μM. (λ_{ex}=488 nm). **(B)** Linear relationship between the fluorescence intensity of FITC-labeled ChOx at 520 nm and the concentration of FITC-labeled ChOx. **(C)** Fluorescence spectra of unloaded FITC-labeled ChOx in the supernatant after formation of FITC-labeled AChE/ChOx-loaded Fe^{3+}-ZIF-90 MOFs (λ_{ex}=488 nm).

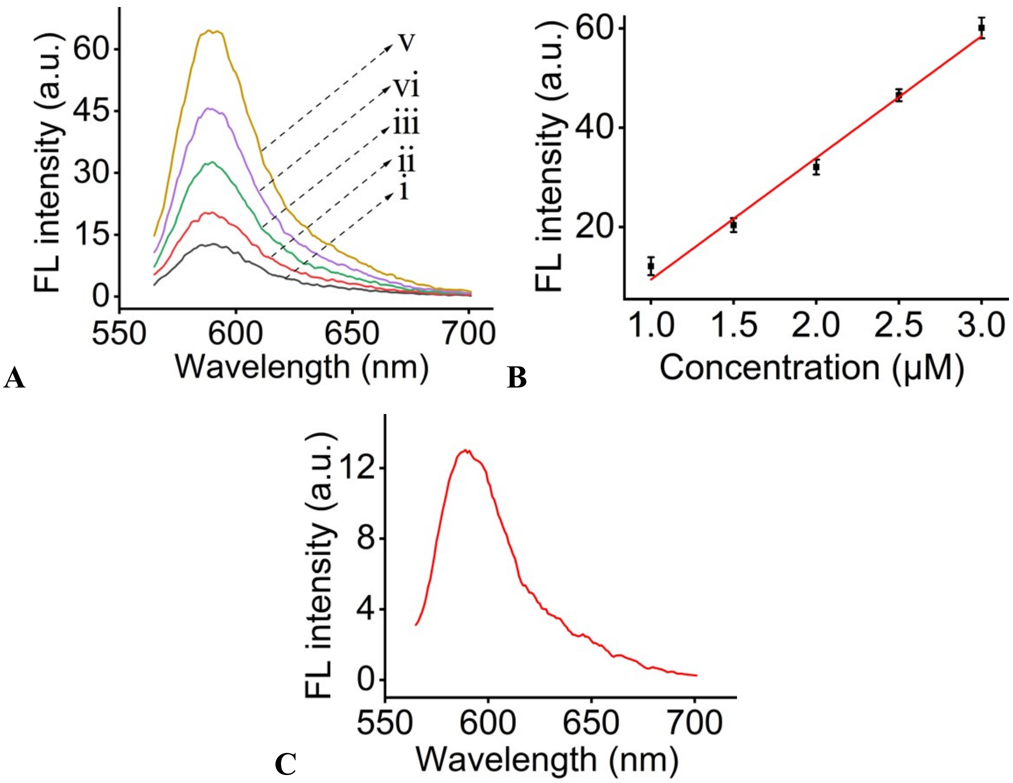

Fig. S13. (A) Fluorescence spectra of Atto 565-labeled AChE at different concentrations: (**i**) 1 μM; (**ii**) 1.5 μM; (**iii**) 2 μM; (**iv**) 2.5 μM; (**v**) 3 μM (λ_{ex}=561 nm). **(B)** Linear relationship between the fluorescence intensity of Atto 565-labeled AChE at 585 nm and the concentration of Atto 565-labeled AChE. (**C**) Fluorescence spectra of unloaded Atto 565-labeled AChE in the supernatant after formation of AChE/ChOx-loaded Fe^{3+}-ZIF-90 MOFs.

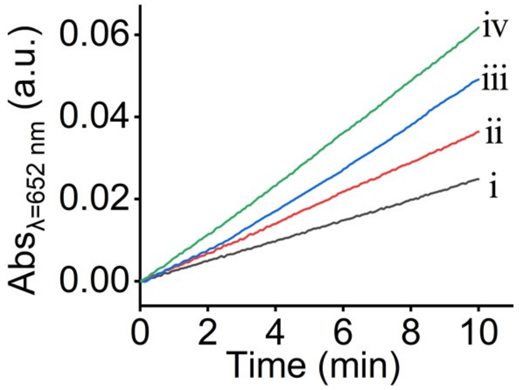

Fig. S14. Time-dependent absorbance changes of $TMB^{\bullet+}$ upon acetylcholine-driving cascade in the presence of AChE/ChOx-loaded Fe^{3+}-ZIF-90 particles with various loading amount of AChE: (**i**) 42.6 μg mg^{-1}; (**ii**) 62.2 μg mg^{-1}; (**iii**) 81.5 μg mg^{-1}; (**iv**) 101.2 μg mg^{-1}. In all experiment, 2 mM of TMB, 10 mM of acetylcholine and 100 μg mL^{-1} AChE/ChOx-loaded Fe^{3+}-ZIF-90 particles were used.

Table S1. Comparison of acetylcholine detection limit of different methods.

Methods	Detection limits (μM)	References
Electrochemistry	5	[4]
Electrochemistry	205	[5]
Electrochemiluminescence	4.7	[6]
Electrochemiluminescence	1.7	[7]
Fluorescence	15	[8]
Fluorescence	50	[9]
Colorimetry	11	[10]
Colorimetry	78	This work

References

1 Y. Qin, Y. Ouyang, J. Wang, X. Chen, Y. S. Sohn and I. Willner, *Nano Lett.*, 2023, **23**, 8664-8673.

2 J. F. Perez-Benito, *J. Phys. Chem. A*, 2004, **108**, 4853-4858.

3 B. Ensing, F. Buda and E. J. Baerends, *J. Phys. Chem. A*, 2003, **107**, 5722-5731.

4 L. Kergoat, B. Piro, D. T. Simon, M. C. Pham, V. Noël and M. Berggren, *Adv. Mater.*, 2014, **26**, 5658-5664.

5 M. L. Colombo, J. V. Sweedler and M. Shen, *Anal. Chem.*, 2015, **87**, 5095-5100.

6 S. Deng, J. Lei, L. Cheng, Y. Zhang and H. Ju, *Biosens. Bioelectron.*, 2011, **26**, 4552-4558.

7 X. F. Wang, Y. Zhou, J. J. Xu and H. Y. Chen, *Adv. Funct. Mater.*, 2009, **19**, 1444-1450.

8 S. Liao, Y. Qiao, W. Han, Z. Xie, Z. Wu, G. Shen and R. Yu, *Anal. Chem.*, 2011, **84**, 45-49.

9 S. Mangalath, S. Abraham and J. Joseph, *Chem. Eur. J.*, 2017, **23**, 11404-11409.

10 P. T. Nguyen, J. Lee, A. Cho, M. S. Kim, D. Choi, J. W. Han, M. I. Kim and J. Lee, *Adv. Funct. Mater.*, 2022, **32**, 2112428.
